# Supplementary material for: Let It Out (LIO) study: protocol for a mixed-methods study to optimise the design and assess the feasibility of an online emotional disclosure-based intervention in UK hospices
Source: BMJ Open. 2021 May 11;11(5):e047135. doi: 10.1136/bmjopen-2020-047135 (PMC8117994; doi:10.1136/bmjopen-2020-047135)
Supplement: Author's manuscript [file bmjopen-2020-047135.draft_revisions.pdf]

BMJ Open is committed to open peer review. As part of this commitment we make the peer review history of every article we publish publicly available.

When an article is published we post the peer reviewers' comments and the authors' responses online. We also post the versions of the paper that were used during peer review. These are the versions that the peer review comments apply to.

The versions of the paper that follow are the versions that were submitted during the peer review process. They are not the versions of record or the final published versions. They should not be cited or distributed as the published version of this manuscript.

BMJ Open is an open access journal and the full, final, typeset and author-corrected version of record of the manuscript is available on our site with no access controls, subscription charges or pay-per-view fees (http://bmjopen.bmj.com).

If you have any questions on BMJ Open's open peer review process please email info.bmjopen@bmj.com

BMJ Open

Let It Out (LIO) study: protocol for a mixed-methods study to optimise the design and assess the feasibility of an online emotional disclosure-based intervention in UK hospices

Journal:	*BMJ Open*
Manuscript ID	bmjopen-2020-047135
Article Type:	Protocol
Date Submitted by the Author:	19-Nov-2020
Complete List of Authors:	McInnerney, Daisy; UCL, Marie Curie Palliative Care Research Department, Division of Psychiatry<br>Candy, Bridget; UCL, Marie Curie Palliative Care Research Department, Division of Psychiatry<br>Stone, Patrick; UCL, Marie Curie Palliative Care Research Department, Division of Psychiatry<br>Kupeli, Nuriye; UCL, Marie Curie Palliative Care Research Department, Division of Psychiatry
Keywords:	PALLIATIVE CARE, PSYCHIATRY, MENTAL HEALTH, STATISTICS & RESEARCH METHODS, QUALITATIVE RESEARCH

SCHOLARONE™
Manuscripts

I, the Submitting Author has the right to grant and does grant on behalf of all authors of the Work (as defined in the below author licence), an exclusive licence and/or a non-exclusive licence for contributions from authors who are: i) UK Crown employees; ii) where BMJ has agreed a CC-BY licence shall apply, and/or iii) in accordance with the terms applicable for US Federal Government officers or employees acting as part of their official duties; on a worldwide, perpetual, irrevocable, royalty-free basis to BMJ Publishing Group Ltd ("BMJ") its licensees and where the relevant Journal is co-owned by BMJ to the co-owners of the Journal, to publish the Work in this journal and any other BMJ products and to exploit all rights, as set out in our licence.

The Submitting Author accepts and understands that any supply made under these terms is made by BMJ to the Submitting Author unless you are acting as an employee on behalf of your employer or a postgraduate student of an affiliated institution which is paying any applicable article publishing charge ("APC") for Open Access articles. Where the Submitting Author wishes to make the Work available on an Open Access basis (and intends to pay the relevant APC), the terms of reuse of such Open Access shall be governed by a Creative Commons licence – details of these licences and which Creative Commons licence will apply to this Work are set out in our licence referred to above.

Other than as permitted in any relevant BMJ Author's Self Archiving Policies, I confirm this Work has not been accepted for publication elsewhere, is not being considered for publication elsewhere and does not duplicate material already published. I confirm all authors consent to publication of this Work and authorise the granting of this licence.

For peer review only

Let It Out (LIO) study: protocol for a mixed-methods study to optimise the design and assess the feasibility of an online emotional disclosure-based intervention in UK hospices

Authors:

Daisy McInnerney[1a], Bridget Candy[1], Patrick Stone[1], Nuriye Kupeli[1]

Affiliations:

[1] Marie Curie Palliative Care Research Department, Division of Psychiatry, University College London, London, UK

Corresponding author:

a. Wing B, Floor 6, Maple House, 149 Tottenham Court Road, London, W1T 7NF; daisy.mcinnerney.18@ucl.ac.uk

Word count: 3998

For peer review only - http://bmjopen.bmj.com/site/about/guidelines.xhtml

ABSTRACT

Introduction: The current COVID-19 pandemic has forced hospices to look for more ways to support people remotely, including psychological support. Emotional disclosure-based interventions hold potential as a way of providing support remotely. However, evidence of their efficacy in people with terminal illness is mixed. Reviews have highlighted this may be due to interventions not being tailored to the unique needs of this population. In response to this, we are developing Let It Out (LIO), an online, self-guided emotional disclosure-based intervention tailored for people living with terminal illness.

Aims: The primary objective of the study is to optimise the design of the LIO intervention. Secondary objectives include assessing its acceptability and feasibility; exploring potential impact on wellbeing; identifying potential adverse effects; and informing choice of outcome measures for potential future evaluation.

Methods and analysis: ≤40 people living with a terminal illness under the care of hospices in England and Scotland will receive the online LIO intervention. LIO consists of three, self-guided expression sessions over two weeks. Outcome measures will include a structured feedback form and validated measures of physical and psychological health collected at baseline, immediately after, and one, four and eight weeks after the final expression session. ≤15 participants will also be invited to take part in a semi-structured interview to further explore their experience and views. ≤30 UK hospice staff, and ≤15 informal carers of participants testing LIO will take part in focus groups and/or semi-structured interviews. A process evaluation to triangulate quantitative and qualitative data will be taken to inform optimisation of the intervention design.

Ethics and dissemination: The study is approved by the University College London Research Ethics Committee (reference: 15281/002). The findings will be shared through peer-reviewed scientific journals and conferences, and traditional, online and social media platforms.

KEYWORDS

PALLIATIVE CARE, PSYCHIATRY, MENTAL HEALTH, STATISTICS & RESEARCH METHODS, QUALITATIVE RESEARCH

For peer review only - http://bmjopen.bmj.com/site/about/guidelines.xhtml

ARTICLE SUMMARY

Strengths and limitations of this study

- The LIO intervention has been developed based on evidence from a scoping review of previous research, and refined drawing on insights from testing it in a slightly different form in another population and feedback from key stakeholders.
- The acceptability and feasibility of the intervention will be explored using a rich, mixed-methods approach, using process evaluation to triangulate quantitative recruitment, retention and acceptability data with qualitative written and interview feedback.
- Recruitment may be restricted by the relatively short recruitment period and potential limitations on hospice staff capacity (due to COVID-19 related pressures on hospice teams).
- The study is taking place during a time of transition and ongoing change in methods of care delivery, necessitated by the evolving COVID-19 pandemic that may restrict generalisability of the findings outside of this context.
- This is a single-arm study not designed to formally evaluate efficacy of the intervention.

For peer review only

INTRODUCTION

People living with terminal illness carry a unique emotional burden and can experience significant distress.(1–4) Psychological support is a key part of the holistic care provided by hospices and other palliative care services.(5–8) But evidence suggests psychological service provision at the end-of-life is currently inadequate, and services are often under-resourced and over-burdened.(9–11) Since the start of the COVID-19 pandemic in early 2020, hospice funding and capacity have been placed under even more threat.(12) Hospices are increasingly turning to telehealth approaches to deliver care to patients.(13) At present this is necessary to help minimise face-to-face contact in line with recommended social distancing practices that are particularly crucial for this vulnerable population.(14) However, delivery of care using such methods is potentially cost- and time-effective, and if found to be beneficial, may endure beyond the resolution of the pandemic to help broaden access to palliative care.

Emotional disclosure (ED)-based therapies, such as expressive writing or talking, are easy to implement as a means of support, with minimal requirement for staff supervision and potential to be delivered online.(15,16) The traditional expressive writing protocol is based on the principle that expressing feelings about personal, traumatic experiences can provide comfort.(17) Indeed, there is evidence that such interventions can provide psychological and physical benefits in healthy populations.(18) Expressive writing has also been adapted, to include, for example disclosing about positive events,(15,19,20) writing or talking about stress from a compassionate stance (21–23) or writing about future goals.(24,25) Writing from a compassionate stance has been shown to reduce negative affect (22,26) and improve mood.(27) However, evidence of benefit of ED-based interventions in palliative populations is more mixed.(28,29) The mixed results may be because there has been a lack of research into developing an ED-based intervention tailored to the specific needs of this population.(28) The underlying causal mechanisms, and thus the outcome measures that may be most suitable to capture any potential benefit of such an intervention in this population, also remain unclear.

Based on this gap in the literature, our group conducted a scoping review of ED-based interventions in people with terminal illness.(29) The review aimed to identify potentially effective characteristics of interventions for this population to inform the development of a tailored ED-based intervention. The review identified 25 relevant primary studies, but found that quantitative evidence was not available to determine which, if any, characteristics may be most effective.(*McInnerney et al., 2020, manuscript under review*) However, the qualitative analysis of authors' experiences of testing the interventions highlighted a number of themes that may be important to consider when developing an ED-based intervention for people with terminal illness. These include flexibility of when, where and how to complete the intervention, clarity of instructions and provision of a safe environment for disclosure.

For peer review only - http://bmjopen.bmj.com/site/about/guidelines.xhtml

Building on the results of this scoping review, we developed an ED-based intervention in collaboration with key stakeholders named Let It Out (LIO). We then refined its design by incorporating insights from a trial we ran of an adapted version of the intervention tested in the general population during the COVID19 pandemic,(30) and further stakeholder feedback. This study represents the next phase of the intervention development process.

Aims and objectives

The primary objective of the study is to optimise the design of an ED-based intervention (LIO) tailored for people receiving palliative care from a UK hospice. The secondary objectives are to:

1. Assess whether the intervention is acceptable to use and feasible to be implemented in UK hospice services.
2. Explore the potential impact of the intervention on psychological and physical wellbeing, and identify any potential adverse effects.
3. Inform the choice of outcome measures and study design for possible future large-scale intervention evaluation.
4. Explore the causal mechanisms underlying potential intervention efficacy.
5. Explore the core concerns of people following referral to hospice care.

METHODS AND ANALYSIS

Design

A multi-site, mixed-methods longitudinal study. The study design has been developed in line with the latest Medical Research Council guidelines,(31,32) which recommend an iterative intervention development and evaluation process. Mixed-methods are recognised as an appropriate way for researchers to approach the development and evaluation of complex interventions in palliative care.(33) In this study, quantitative measures of feasibility, acceptability and possible impact, will be integrated through process evaluation with qualitative interviews and focus groups. These qualitative data will capture more nuanced feedback on the design, acceptability and perceived effect of the intervention that may be missed through quantitative assessment alone.(33,34)

Setting

The study will take place in ≤10 UK hospice services across England and Scotland. A list of participating sites is available from the authors.

For peer review only - http://bmjopen.bmj.com/site/about/guidelines.xhtml

Participants

The study will be conducted in three population groups recruited from participating hospices:

Group 1: ≤40 adults living with terminal illness receiving palliative care from a UK hospice (on an inpatient, day care, outpatient or community basis). A sample size of 40 is recognised by the National Institute of Health Research as being appropriate for intervention feasibility and pilot work that is not designed to evaluate efficacy.(35,36)

Group 1 exclusion criteria

- Not expected to survive for longer than ten weeks (based on clinical judgement).
- Lack of capacity to give informed consent or may be expected to lose capacity to give informed consent within ten weeks (based on clinical judgement).
- Known diagnosis or history of severe depression, suicidal tendencies, psychotic symptoms, severe mood disorders, mania, schizophrenia, psychopathic/borderline personality disorder, severe and episodic (Type 1) bipolar (affective) disorder (that is not well controlled).
- Not able to understand and communicate in English.

Group 2: ≤15 informal (adult friend or family) carers of Group 1 participants.

Group 3: ≤30 paid or voluntary hospice staff involved in organisation, delivery or implementation of psychological and/or emotional support.

Recruitment

Group 1

Clinical and research staff at participating hospice sites will identify participants who may be eligible to take part in the study between October 2020 and January 2021. They will make initial contact, explain the study and if the participant is interested, provide them with an invitation letter, email or link to the study website. The study website hosts the participant information sheet and a link to an online reply form. Participants can indicate on the reply form if they would or would not like to take part.

If participants select yes, they can progress to the online informed consent and advance consent form, which asks what participants would like to happen to their data if they lose capacity over the course of their participation in the study. If they select no, they have the option to provide their reasons for not taking part. There is also an option to request further information from the research team before making a decision. If

For peer review only

For peer review only - http://bmjopen.bmj.com/site/about/guidelines.xhtml

participants select this option, they are prompted to provide their contact details and a member of the research team will phone or email them to discuss the study further. If, having discussed the study, participants choose to take part, they can progress to the online informed consent and advance consent forms. The research team will inform the relevant hospice when a participant from their service consents to take part, so their participation can be recorded on their patient notes. The participant will be reminded of their right to withdraw from the study at any time throughout the study in online materials and through direct communication with the research team.

Group 2 (informal carers) will be recruited through researcher contact with Group 1 participants. If their carer indicates they may be interested in taking part in an interview, the research team will arrange a time to discuss the study; if interested in taking part, they will be sent a link to the online participant information sheet and informed consent form.

Group 3 (staff and volunteers) will be recruited through liaison with clinical leads at each hospice site. The research team will approach eligible staff members to introduce the study, and if interested, provide them with a link to the online participant information sheet and informed consent form.

The study will be presented at hospice staff team meetings and internal newsletters to support recruitment.

The intervention

Development

The Let It Out (LIO) intervention has been developed in collaboration with a group of stakeholders, including clinical and health psychologists, a psychiatrist, palliative care consultants, a hospice supportive care service manager, palliative care specialist researchers and PPI representatives. The intervention was first developed based on the findings of a scoping review conducted by the research team that explored potentially effective characteristics, theoretical underpinnings, and facilitators and barriers of studying and implementing existing ED-based interventions in people with terminal illness.(29) Based on the review findings, the proposed LIO intervention is flexible in terms of when, where and how it can be completed; provides clear, structured instructions; and emphasises the importance of completing sessions in a safe, comfortable setting. During the COVID-19 pandemic, the research team adapted the intervention to test its effect on distress in the general population during the pandemic (the LIO-C study).(30) Based on that study and feedback from participants, adaptations were made to the LIO intervention to minimise any potential risk of causing short-term distress; namely, by incorporating clearer directions on how to express feelings with self-compassion.

For peer review only - http://bmjopen.bmj.com/site/about/guidelines.xhtml

Theoretical basis

The intervention development process has been guided by emotion regulation theory, which posits that the active component of ED-based interventions is the emotional arousal following expression of emotions.(37–39) This account suggests that one is able to develop acceptance of their thoughts and feelings through mastery, self-efficacy and control of their emotions. This account also sits within theories underlying positive psychology and self-compassion interventions,(19,40) which propose that one is able to gain a better understanding of the emotions associated with their experiences by learning how to more helpfully structure thoughts and feelings. We recognise that there is unlikely to be a single process underlying ED; indeed, several alternative processes have been proposed to explain ED including emotional inhibition, cognitive adaptation and exposure.(41–44) One aim of this study, in line with the MRC complex intervention development guidance,(32) is to explore the mechanisms through which LIO may be effective and inform understanding of its potentially active components.

Outline

LIO is a self-guided intervention designed to enable people living with terminal illness to self-compassionately express their feelings in a way that may help to bring them comfort. In this study, participants will be sent a link to online intervention instructions, as well as a PDF intervention guide that can be downloaded from the online survey platform. The guide provides step-by-step instructions for participants to work through the study and intervention. Over two weeks, participants are asked to express their feelings in a self-compassionate way in response to three prompts in three separate ‘expression sessions’ (one prompt per session). Figure 1 presents the general instructions given to participants to use across each session and the prompts for each session.

Figure 1. **General instructions and session specific prompts as part of LIO guide (written version)**

Each session is 20-30 minutes and participants are advised to complete them in a comfortable place, and, if possible, in private (although it is recognised this may not be feasible or wanted for all participants). Participants can either handwrite, type or audio-record (using their personal mobile phone or laptop) their response to the prompts. There are two guides, both available for download: the first for those choosing to write, the second for those choosing to audio-record. The full guides are available on reasonable request from co-authors.

To make the intervention as accessible as possible, it is acceptable for participants to have support from a carer to help complete the expression sessions, and the associated questionnaires. This help can be delivered in any way that works best for the participant. Participants have the option to share their responses to each expression session with the research team, or to keep them private, depending on personal preference. The

For peer review only - http://bmjopen.bmj.com/site/about/guidelines.xhtml

guide and online platform also includes signposts to other helpful resources that can support mental wellbeing. In addition, the guide provides a space for participants to plan when and where they will complete each intervention session, based on the theory of action-planning to help bridge the intention-behaviour gap.(45)

Data collection

Overview

The participant timeline is summarised in Table 1. Group 1 participants will complete online self-report questionnaires at baseline (prior to receiving the intervention) and one week, four weeks and eight weeks after the final intervention session, using the REDCap online survey platform.(46,47) Group 1 participants will also be prompted to log in to the platform during or after each expression session to indicate they have completed the session, and where they will have the option to share their response with the research team. Directly after each expression session they will also be prompted to complete a short self-report questionnaire to monitor their mood, and their perception of how meaningful and personal the session was. Researchers will contact each participant by phone or email (participant preference) at the end of the first and second week of the intervention to check wellbeing and answer any questions. ≤15 Group 1 participants will also be invited to complete an online audio-recorded interview with a researcher within the eight week follow-up period to explore their experiences and feedback in more detail than captured in the feedback form. If during contact with the participant the research team suspects they may no longer have capacity for informed consent, or is experiencing severe distress, the hospice clinical team will be informed and capacity formally assessed. If the participant is deemed no longer eligible to take part in the study, they will be withdrawn and the directives from their advance consent form followed.

For peer review only

For peer review only - http://bmjopen.bmj.com/site/about/guidelines.xhtml

Table 1. Participant timeline for Group 1 participants

Measures/ data collected	Baseline	2 week intervention period (expression sessions)			Weeks post-intervention			
		1	2	3	1	4	8	Within 8 (n≤15)
Demographics	X							
IPOS	X			X	X	X	X	
PHQ-9	X			X	X	X	X	
GAD-7	X			X	X	X	X	
SQS	X			X	X	X	X	
Uptake of existing mental health services	X			X	X	X	X	
Expression session 1		X						
Expression session 2			X					
Expression session 3				X				
Meaningfulness / personal / mood rating		X	X	X				
Feedback form				X				
Semi-structured interview								X

Abbreviations: GAD-7, Generalised Anxiety Disorder-7; IPOS, Integrated Palliative Care Outcome Scale; PHQ-9, Patient Health Questionnaire-9; SQS, Single-item Sleep Quality Scale

For peer review only - http://bmjopen.bmj.com/site/about/guidelines.xhtml

Expression sessions

Over the course of the two week intervention, participants will type, handwrite or audio-record (format chosen by the participant) their response to the three prompts. Handwritten and audio-recorded responses will be transcribed for analysis. It is not mandatory for participants to share their expression session responses if they would prefer to keep them private. Methods for data collection and transfer are summarised in Table 2.

Table 2. Methods of expression session data collection and transfer for Group 1 participants

Method of disclosure	Data collection	Method for transfer to research team
Typed into platform	Participant types directly into text box on online survey platform (REDCap via Data Safe Haven).	Research team have access to data entered onto REDCap via Data Safe Haven.
Typed on device	Participant types into word processor on own device.	Participant saves file on own device and uploads it to the REDCap via Data Safe Haven survey platform.
Hand-written	Participant handwrites responses on own paper.	Participant takes photo of handwritten responses using mobile phone camera and sends via WhatsApp to research team* (end-to-end encryption). Photos transferred to Data Safe Haven and deleted from phone.
Audio-recorded on smart phone	Participant records themselves using either inbuilt recording / notes app on their phone or the Rev app (free to download).	Participant sends audio-recording via WhatsApp to research team* (end-to-end encryption). Audio-recording transferred to Data Safe Haven and deleted from phone.
Audio-recorded – on laptop/computer	Participant records themselves using free Audacity software.	Participant saves file on own device and uploads it to the REDCap via Data Safe Haven survey platform.

*The research team will have a dedicated study phone that a member of the research team keeps only on their person during working hours (9am – 5pm Monday – Friday) and is kept in a secure locked cabinet outside of those hours. The phone will be checked once a day for recordings or photos received, at which point the recording will be transferred to the dedicated project file on Data Safe Haven and deleted from the phone.

For peer review only - http://bmjopen.bmj.com/site/about/guidelines.xhtml

Measures

1. Demographics

The following data will be collected via an online self-report questionnaire at baseline: approximate date of hospice referral, age, gender, ethnicity, marital status, religious background, level of education, type of care (eg, inpatient, outpatient, day care), diagnosis, mental health history and COVID19 status.

2. Self-report measures at baseline and immediately, 1-week, 4-weeks and 8-weeks post-final expression session

The following will be measured as preliminary indicators of intervention effect on wellbeing, to provide insights into potential causal mechanisms of the intervention, and to inform potential outcome measure selection for future research:

- *Integrated Palliative Care Outcome Scale (IPOS) – patient version* (48) – an updated, brief (10 questions, 17 items) version of the POS instrument. IPOS was developed specifically to capture a range of the most important concerns of people living with palliative-stage disease, covering symptoms, information needs, practical concerns, anxiety or low mood, family anxieties and overall feeling of being at peace. Items in question 2 to 9 are scored from 0 to 4, and overall scores can range from 0 to 68. Questions 1 and 10 are not scored.
- *Patient Health Questionnaire (PHQ) 9* (49) - a 9-item instrument developed to screen for major depressive disorder, and validated in palliative care populations (50). Items are scored 0 to 3, and it can be scored continuously or with validated cut-offs for mild (5–9), moderate (10–14), moderately severe (15–19) and severe depression (20–27) (51,52). The cut-off for a depression "case" is 10.
- *Generalised Anxiety Disorder (GAD) 7* (53) – a validated 7-item anxiety screening instrument that is used to screen for anxiety in palliative care settings. Items are scored 0 to 3, and total scores range from 0 to 21. Severity of anxiety symptoms can be classed as None (0-4), Mild (5-9), Moderate (10-14) or Severe (15-21). The cut-off score for a Generalised Anxiety Disorder "case" is 10.
- *Single-item Sleep Quality Scale (SQS)* (54) – a short, pragmatic, validated tool for assessing sleep quality in clinical populations, selected to reduce burden on participants from lengthier alternatives such as the Pittsburgh Sleep Quality Index (PSQI) (55). A single visual analogue scale asks respondents to rate their overall quality of sleep over the last seven days on a scale of 0 to 10. Scores are used to categorise sleep quality as: Terrible (0), Poor (1-3), Fair (4-6), Good (7-9) and Excellent (10).
- *Uptake of existing mental health services* – a three-item form developed for this study asking participants if they have (1) ever sought help from a healthcare professional for their mental health;

For peer review only - http://bmjopen.bmj.com/site/about/guidelines.xhtml

(2) sought help from a healthcare professional for their mental health over the past two weeks; and (3) any plans to seek help from a healthcare professional for their mental health in the future. Response options are 'Yes', 'No', or 'Prefer not to say'.

3. Measures immediately after each expression session

A 3-item, 7-point Likert scale based on a measure used in a study testing a similar intervention (56), measuring how personal and meaningful participants' felt their expression session was (not very personal/not very meaningful (-3) to (+3) very personal/very meaningful). The last item will be a mood check where participants are asked to rate how they feel on a 7-point scale (very sad (-3) to (+3) very happy).

4. Measure after final expression session

Participants will be asked to complete an online feedback form exploring their experience of completing the intervention, if they needed any assistance from a carer and the nature of that assistance, and their fidelity to the protocol. The feedback form questions were informed by work on ethical research in vulnerable populations (57) and The Theoretical Framework of Acceptability for healthcare system interventions (58).

Qualitative interviews

Semi-structured interviews (n≤15) will be conducted after the participant has completed all three sessions of the intervention; or, if the participants do not complete all three sessions, after they complete their final session. Interviews will explore participants' experience of the intervention and questionnaires, including whether they experienced any negative feelings during or after each expression, whether they found the intervention helpful, and the appropriateness and relatability of the expression session prompts, expanding on the topics covered in the feedback form.

Semi-structured interviews will also be conducted with informal carers of Group 1 participants after they have completed their final expression session. Focus groups or semi-structured interviews (depending on practicalities of arranging a suitable time) with hospice staff and volunteers will also be conducted. All interviews and focus groups will take place virtually using Microsoft Teams to record the sessions to minimise face-to-face interactions in line with government COVID-19 guidance. Recordings will be transcribed for analysis.

For peer review only - http://bmjopen.bmj.com/site/about/guidelines.xhtml

Data analysis

Primary objective: process evaluation and intervention proposal

Using the qualitative and quantitative data collected from Groups 1, 2 and 3 (and in combination with evidence that has informed this research protocol from prior work (reviews and a trial) conducted by the research team), a triangulation approach will be applied drawing on process evaluation methodology.(34,59) Process evaluation is recognised as a valuable method to optimise intervention design and inform evaluation during feasibility testing phases.(34) Through the process evaluation, we aim to develop a preliminary theoretical model to inform understanding of potential causal mechanisms of LIO and any contextual and implementation factors that may be associated with any potential variation in outcomes. Insights from the process evaluation will inform the final intervention proposal. Analysis will be led by DM in iterative consultation with the research team and advisory group.

Secondary objectives

Feasibility and acceptability

Due to the preliminary nature of this study, no pre-specified criteria have been defined to establish acceptability of the LIO intervention, or the feasibility of the study procedures. The following measures of feasibility and acceptability will be analysed:

A. Retention

i. Percentage of participants recruited completing each data collection time-point.

B. Adherence

i. Percentage of participants completing all three expression sessions (a) within 20-30 minute time frame and (b) in a private space.
ii. Blinded independent assessor review of texts to determine which prompt was being responded to.
iii. Linguistic Inquiry Word Count (LIWC) analysis of expression session texts/transcripts (60) to analyse percentages of emotion, self and prompt-related words. LIWC is a computer programme that calculates word count, and percentages of words used that reflect different emotions, thinking styles and concerns.

C. Acceptability

i. Descriptive quantitative analysis of responses to the feedback form.

For peer review only - http://bmjopen.bmj.com/site/about/guidelines.xhtml

ii. Qualitative thematic analysis of free-text responses to feedback form and interviews with patient, carer and staff participants guided by The Theoretical Framework of Acceptability for healthcare system interventions.(58)

Impact on psychological and physical wellbeing

A. Thematic analysis of interviews and feedback from free-text responses from patients, carer and staff participants.
B. Descriptive pre-post analysis of changes in outcome measures (IPOS, HADS, GAD7, SQS, uptake on mental health services).
C. Descriptive summary of number of adverse effects reported by clinical team and participants.

Choice of outcome measure: inferences of potentially suitable outcome measures will be based on results from descriptive pre-post analysis of outcome measures, and qualitative analysis of interviews and feedback from free-text responses from patients, carers and staff participants regarding reported effects of the intervention.

Potential underlying mechanisms: responses to expression session prompts will be analysed quantitatively using LIWC software (60) and triangulated with indicators of intervention impact on wellbeing to inform development of a preliminary theoretical model.

Core concerns on referral to hospice: responses to expression session prompts will be analysed using inductive thematic analysis.

Patient and Public Involvement (PPI)

The funding application for this programme of work was reviewed by Mr Peter Buckle, a Marie Curie PPI representative. Peter is also a member of the advisory group overseeing the conduct of the PhD studentship of which this study is a part. Peter, along with a second PPI representative (Dori-Anne Finlay) have reviewed the study and intervention design. Peter and Dori-Anne will be consulted at key stages of the research. Furthermore, the study as a whole has been designed based on the principles of co-design; the data from participants will be used to inform the design of the final intervention and potential future research.

Data management

All survey-based data (demographics and health-related questionnaires) will be collected using REDCap (46,47) via University College London Data Safe Haven. The Data Safe Haven has been certified to the ISO27001 information security standard and conforms to NHS Digital's Information Governance Toolkit. Built

For peer review only

For peer review only - http://bmjopen.bmj.com/site/about/guidelines.xhtml

using a walled garden approach, the data is stored, processed and managed within the security of the system, avoiding the complexity of assured endpoint encryption. A file transfer mechanism enables information to be transferred into the walled garden simply and securely. A data management plan has been registered and approved by the University College London Data Protection Office outlining methods to maintain participant privacy and data integrity and confidentiality. At the end of the project, anonymised data will be uploaded to open-access data repository ReShare.(61)

ETHICS AND DISSEMINATION

Confidentiality

All participant data will be collected via the secure REDCap via Data Safe Haven platform,(46,47) or Microsoft Teams (interviews/focus groups), except audio-recordings or photographs of expression sessions, which will be sent directly to a dedicated study telephone via WhatsApp secured by end-to-end encryption. The phone will be carried by a member of the research team or stored in a locked drawer. Only members of the research team will have access to participant data. Consent and data collection forms will be securely stored in the University College London Data Safe Haven. During analysis, data will be pseudonymised using a unique ID that could not be linked to a participant's identity by anyone outside of the research team. Data will be anonymised 3-months after participants' involvement in the study ends.

Serious Adverse Events (SAE)

Any SAEs attributed to a person's participation in the study will be recorded on the online study platform, and in the participant's medical records. SAE forms will be provided by the chief investigator to the sponsor within five days of becoming aware of the event.

Dissemination

The findings of this study will be disseminated through peer-reviewed scientific journals, conferences, and traditional, online and social media.

ACKNOWLEDGEMENTS

The LIO study team are grateful to the group of advisors who have provided invaluable feedback on the design of the LIO intervention and study design to date: Peter Buckle, Jason Davidson, Dori-Anne Finlay, Dr Syd Hiskey, Dr Joana Johnson, Prof. Marc Serfaty, Dr Nick Troop. We are also grateful to the research and clinical leads and supporting staff at participating sites for their feedback on the intervention and study design, and support through the research governance process, particularly: Dr Anne Finucane, Dr Emma

For peer review only - http://bmjopen.bmj.com/site/about/guidelines.xhtml

Carduff, Dr John MacArtney, Dr Ami Nwosu, Dr Adrian Tookman, Gail Holloway, Rachel Perry. Thank you also to Dr Sabine Best and Dr Briony Hudson for support in facilitating multi-site research across Marie Curie hospice sites.

AUTHOR CONTRIBUTIONS

The study and intervention design was led by DM. NK, BC and PS have collaborated at all stages on study and intervention development. NK is the Chief Investigator, based at University College London. DM is the PhD student managing the project. DM led the writing of this manuscript. All authors contributed to and approved the final draft.

FUNDING

This work was supported by Marie Curie (MCCC-FCH-18-U) and Economic and Social Research Council collaborative funding (ES/P000592/1). This work was also supported by core funding by Marie Curie (grant number: MCCCFPO-16-U) and an Alzheimer's Society (AS) funded fellowship awarded to NK (grant number: 399 AS-JF-17b-016).

COMPETING INTERESTS STATEMENT

None declared

REFERENCES

1. Galfin JM, Watkins ER. Construal level, rumination, and psychological distress in palliative care. *Psychooncology*. 2012 Jun;21(6):680–3.

2. Kolva E, Rosenfeld B, Pessin H, et al. Anxiety in Terminally Ill Cancer Patients. *J Pain Symptom Manage.* 2011 Nov;42(5):691–701.

3. Okuyama T, Endo C, Seto T, et al. Cancer patients' reluctance to discuss psychological distress with their physicians was not associated with underrecognition of depression by physicians: a preliminary study. *Palliat Support Care*. 2009 Jun;7(2):229–33.

4. Wilson KG, Chochinov HM, Skirko GM, et al. Depression and Anxiety Disorders in Palliative Cancer Care. *J Pain Symptom Manage*. 2007 Feb;33(2):118–29.

5. Ross K, Dexter-Smith, S. Psychological best practice in inpatient services for older people. *The British Psychological Society.* 2017. Available from: https://shop.bps.org.uk/psychological-best-practice-in-inpatient-services-for-older-people (accessed 15 November 2020).

For peer review only - http://bmjopen.bmj.com/site/about/guidelines.xhtml

For peer review only

6. Cherny NI, on behalf of the ESMO Guidelines Working Group. ESMO Clinical Practice Guidelines for the management of refractory symptoms at the end of life and the use of palliative sedation. *Ann Oncol*. 2014 Sep 1;25(suppl 3):iii143–52.

7. Connor SR, Sepulveda Bermedo MC. Global atlas of palliative care at the end of life. *Worldwide Palliative Care Alliance, World Health Organization*. 2014. Available from: https://www.who.int/nmh/Global_Atlas_of_Palliative_Care.pdf (accessed 15 November 2020).

8. Kalus C, Beloff H, Brennan J, McWilliams E, et al. The role of psychology in end of life care. *The Professional Board of the British Psychological Society*. 2008. Available from: https://www.palliativpsychologie.de/wp-content/uploads/Kalus_2008_The-Role-of-Psychology-in-End-of-Life-Care.pdf (accessed 15 November 2020).

9. Atkin N, Vickerstaff V, Candy B. 'Worried to death': the assessment and management of anxiety in patients with advanced life-limiting disease, a national survey of palliative medicine physicians. *BMC Palliat Care*. 2017 Dec;16(1).

10. Hospice UK. Impact of cost pressures - findings April 2019. 2019. *Hospice UK*. Available from: https://www.hospiceuk.org/policy-advocacy/briefings-consultations?page=2 (accessed 15 November 2020).

11. Russell C, Fountain A. Role of clinical psychology in UK hospices. *BMJ Support Palliat Care*. 2018 Oct 3;bmjspcare-2018-001594.

12. NHS. Funding arrangements for bed and community care capacity in the hospice sector. 2020. Available from: https://www.england.nhs.uk/coronavirus/wp-content/uploads/sites/52/2020/04/CO365-grant-funding-arrangements-for-the-hospice-sector.pdf (accessed 15 November 2020).

13. Tavabie S, Ball K. Perspectives on dying from COVID-19: Waiting for the storm. *BMJ Support Palliat Care*. 2020. Available from: https://blogs.bmj.com/spcare/2020/03/26/perspectives-on-dying-from-covid-19-waiting-for-the-storm/ (accessed 15 November 2020).

14. Public Health England. Guidance on shielding and protecting people who are clinically extremely vulnerable from COVID-19. *GOV.UK.* 2020. Available from: https://www.gov.uk/government/publications/guidance-on-shielding-and-protecting-extremely-vulnerable-persons-from-covid-19/guidance-on-shielding-and-protecting-extremely-vulnerable-persons-from-covid-19 (accessed 15 November 2020).

15. Baikie KA, Geerligs L, Wilhelm K. Expressive writing and positive writing for participants with mood disorders: An online randomized controlled trial. *J Affect Disord.* 2012;10.

16. Sayer NA, Noorbaloochi S, Frazier PA, et al. Randomized Controlled Trial of Online Expressive Writing to Address Readjustment Difficulties Among U.S. Afghanistan and Iraq War Veterans. *J Trauma Stress*. 2015.28(5):381-90. doi: 10.1002/jts.22047.

17. Pennebaker JW, Beall SK. Confronting a Traumatic Event: Toward an Understanding of Inhibition and Disease. *Journal of Abnormal Psychology*.1986.95(3):274-81. doi.org/10.1037/0021-843X.95.3.274

18. Frattaroli J. Experimental disclosure and its moderators: A meta-analysis. *Psychol Bull*. 2006;132(6):823–65.

For peer review only - http://bmjopen.bmj.com/site/about/guidelines.xhtml

19. Burton CM, King LA. The health benefits of writing about intensely positive experiences. *J Res Personal*. 2004 Apr;38(2):150–63.

20. Niles AN, Byrne Haltom KE, Lieberman MD, et al. Writing content predicts benefit from written expressive disclosure: Evidence for repeated exposure and self-affirmation. *Cogn Emot.* 2016 Feb 17;30(2):258–74.

21. Imrie S, Troop NA. A pilot study on the effects and feasibility of compassion-focused expressive writing in Day Hospice patients. *Palliat Support Care.* 2012 Jun;10(2):115–22.

22. Przezdziecki A, Sherman KA. Modifying Affective and Cognitive Responses Regarding Body Image Difficulties in Breast Cancer Survivors Using a Self-Compassion-Based Writing Intervention. *Mindfulness*. 2016 Oct 1;7(5):1142–55.

23. Sherman KA, Przezdziecki A, Alcorso J, et al. Reducing Body Image-Related Distress in Women With Breast Cancer Using a Structured Online Writing Exercise: Results From the My Changed Body Randomized Controlled Trial. *J Clin Oncol*. 2018 Jul 1;36(19):1930–40.

24. King LA. The Health Benefits of Writing about Life Goals. *Pers Soc Psychol Bull.* 2001 Jul;27(7):798–807.

25. Troop N, Chilcot J, Hutchings L et al. Expressive writing, self-criticism, and self-reassurance. *Psychol Psychother Theory Res Pract*. 2013;86:374–86.

26. Leary MR, Tate EB, Adams CE, et al. Self-compassion and reactions to unpleasant self-relevant events: The implications of treating oneself kindly. *J Pers Soc Psychol.* 2007;92(5):887–904.

27. Odou N, Brinker J. Exploring the Relationship between Rumination, Self-compassion, and Mood. *Self Identity.* 2014 Jul 4;13(4):449–59.

28. Kupeli N, Chatzithedorou G, Troop N, et al. Expressive writing as a therapeutic intervention for people with advanced disease: A systematic review. *BMC Palliat Care.2019;*18(65). doi.org/10.1186/s12904-019-0449-y

29. McInnerney D, Kupeli N, Stone P, et al. Emotional disclosure as a therapeutic intervention in palliative care: a scoping review protocol. *BMJ Open*. 2019;9:e031046. doi: 10.1136/bmjopen-2019-031046.

30. McInnerney D, Kupeli N. Let It Out (LIO) and COVID19: Testing an Online Emotional Disclosure-based Intervention During the COVID19 Pandemic [Internet]. Clinicaltrials.gov. 2020. Available from: https://clinicaltrials.gov/ct2/show/NCT04386668 (accessed 15 November 2020).

31. Craig P, Dieppe P, Macintyre S, et al. Developing and evaluating complex interventions. 2006. *Medical Research Council.* Available from: https://mrc.ukri.org/documents/pdf/complex-interventions-guidance/ (accessed 15 November 2020).

32. O'Cathain A, Croot L, Duncan E, et al. Guidance on how to develop complex interventions to improve health and healthcare. *Open Access.* 2019;(e029954):9.

For peer review only - http://bmjopen.bmj.com/site/about/guidelines.xhtml

33. Farquhar M, Ewing G, Booth S. Using mixed methods to develop and evaluate complex interventions in palliative care research. *Palliat Med*. 2011 Dec;25(8):748-57. doi: 10.1177/0269216311417919

34. Moore GF, Audrey S, Barker M, et al. Process evaluation of complex interventions: Medical Research Council guidance. *BMJ.* 2015;350:h1258

35. Thabane L, Ma J, Chu R, et al. A tutorial on pilot studies: the what, why and how. *BMC Med Res Methodol.* 2010 Jan 6;10(1):1.

36. Hooper R. Justifying sample size for a feasibility study. *National Institute for Health Research*. 2019. Available from: https://www.nihr.ac.uk/documents/nihr-research-for-patient-benefit-rfpb-programme-guidance-on-applying-for-feasibility-studies/20474 (accessed 15 November 2020).

37. Lepore SJ, Greenberg MA, Bruno M, et al. The writing cure: How expressive writing promotes health and emotional well-being. In: The writing cure: How expressive writing promotes health and emotional well-being. Washington, DC, US: American Psychological Association; 2002. p. xii, 313.

38. Cameron LD, Jago L. Emotion regulation interventions: A common-sense model approach. *Br J Health Psychol.* 2008;13(2):215–21.

39. Koole SL. The psychology of emotion regulation: An integrative review. *Cogn Emot.* 2009 Jan 1;23(1):4–41.

40. Quoidbach J, Mikolajczak M, Gross, JJ. Positive interventions: An emotion regulation perspective. *Psychol Bull.* 2015; 141(3):655-93. doi: 10.1037/a0038648.

41. Nicholls S. Beyond Expressive Writing: Evolving Models of Developmental Creative Writing. *J Health Psychol.* 2009 Mar;14(2):171–80.

42. Smyth JM, Pennebaker JW. Exploring the boundary conditions of expressive writing: In search of the right recipe. *Br J Health Psychol.* 2008 Feb;13(1):1–7.

43. Sloan DM, Marx BP. Taking Pen to Hand: Evaluating Theories Underlying the Written Disclosure Paradigm. *Clin Psychol Sci Pract.* 2004;11(2):121–37.

44. Pennebaker JW. Theories, Therapies, and Taxpayers: On the Complexities of the Expressive Writing Paradigm. *Clin Psychol Sci Pract.* 2004;11(2):138–42.

45. Hagger M, Luszczynska A. Implementation intention and action planning interventions in health contexts: state of the research and proposals for the way forward. *Appl Psychol Health Well-Being.* 2014 Mar;6(1):1–47.

46. Harris PA, Taylor R, Minor BL, et al. The REDCap consortium: Building an international community of software platform partners. *J Biomed Inform.* 2019 Jul 1;95:103208.

47. Harris P, Taylor R, Thielke R, et al. Research electronic data capture (REDCap) - A metadata-driven methodology and workflow process for providing translational research informatics support. *J Biomed Inf.* 2009;42(2):377–81.

For peer review only

For peer review only - http://bmjopen.bmj.com/site/about/guidelines.xhtml

48. Murtagh FE, Ramsenthaler C, Firth A, et al. A brief, patient- and proxy-reported outcome measure in advanced illness: Validity, reliability and responsiveness of the Integrated Palliative care Outcome Scale (IPOS). *Palliat Med.* 2019 Sep;33(8):1045–57.

49. Kroenke K, Spitzer RL, Williams JBW, et al. The Patient Health Questionnaire Somatic, Anxiety, and Depressive Symptom Scales: a systematic review. *Gen Hosp Psychiatry.* 2010 Jul;32(4):345–59.

50. Chilcot J, Rayner L, Lee W, et al. The factor structure of the PHQ-9 in palliative care. *J Psychosom Res.* 2013 Jul 1;75(1):60–4.

51. Kroenke K, Spitzer RL, Williams JBW. The PHQ-9. *J Gen Intern Med.* 2001 Sep;16(9):606–13.

52. Spitzer RL, Kroenke K, Williams JBW, and the Patient Health Questionnaire Primary Care Study Group. Validation and Utility of a Self-report Version of PRIME-MDThe PHQ Primary Care Study. *JAMA.* 1999 Nov 10;282(18):1737–44.

53. Spitzer RL, Kroenke K, Williams JBW, et al. A Brief Measure for Assessing Generalized Anxiety Disorder: The GAD-7. *Arch Intern Med.* 2006 May 22;166(10):1092–7.

54. Snyder E, Cai B, DeMuro C, et al. A New Single-Item Sleep Quality Scale: Results of Psychometric Evaluation in Patients With Chronic Primary Insomnia and Depression. *J Clin Sleep Med JCSM Off Publ Am Acad Sleep Med.* 2018 Nov 15;14(11):1849–57.

55. Buysse D, Reynolds C, Monk T, et al. The Pittsburgh Sleep Quality Index: a new instrument for psychiatric practice and research. Psychiatry Res. 1989 May;28(2):193-213. doi: 10.1016/0165-1781(89)90047-4.

56. Kupeli N, Schmidt UH, Campbell IC, et al. The impact of an emotionally expressive writing intervention on eating pathology in female students. *Health Psychol Behav Med.* 2018 Jan 1;6(1):162–79.

57. Sque M, Walker W, Long-Sutehall T. Research with bereaved families: A framework for ethical decision-making. *Nurs Ethics*. 2014 Dec 1;21(8):946–55.

58. Sekhon M, Cartwright M, Francis JJ. Acceptability of healthcare interventions: an overview of reviews and development of a theoretical framework. *BMC Health Serv Res.* 2017 Jan 26;17(1):88.

59. Moore G, Audrey S, Barker M, et al. Process evaluation of complex interventions: UK Medical Research Council (MRC) guidance. MRC Population Health Science Research Network. Available from: https://mrc.ukri.org/documents/pdf/mrc-phsrn-process-evaluation-guidance-final/(accessed 15 November 2020).

60. Tausczik YR, Pennebaker JW. The Psychological Meaning of Words: LIWC and Computerized Text Analysis Methods. *J Lang Soc Psychol.* 2010 Mar;29(1):24–54.

61. UK Data Service. ReShare: archiving and sharing research data. Available from: https://reshare.ukdataservice.ac.uk/ (accessed 15 November 2020).

For peer review only - http://bmjopen.bmj.com/site/about/guidelines.xhtml

For peer review only

For peer review only - http://bmjopen.bmj.com/site/about/guidelines.xhtml

Expression Session topics

- Follow the general instructions in the yellow box below for each Expression Session.
- The topics for each of the three Expression Sessions are described on the next page. Feel free to change the order or repeat a session of your choice if you wish. These topics are just a guide.
- At the beginning of each session, set your timer and try to write in response to the suggested topic for at least 20 minutes. If you become too tired to continue, feel free to pause and pick back up again when you feel able. If at the end of 20 minutes you feel you have more to write, set the timer for another 10 minutes and keep going until you feel ready to stop. We would not recommend you write for longer than 30 minutes in total though.

General Instructions

Keep these in mind throughout each Expression Session

- As you are writing, try to remember and describe the thoughts and emotions you felt during each experience.
- Some of the moments you describe might be painful or difficult to remember. As you describe these experiences, try to write from the perspective of someone who is accepting and understanding of your emotions, and consider how other people may feel the same way when in similar situations. Try to write with kindness and concern for yourself in mind. It might help to think about what you would say to a friend if they were in your shoes, or what a friend might say to you.
- At the end of each session, try to finish by writing a few sentences focussing on expressing warmth and kindness for yourself and your feelings.

Don't worry about spelling, sentence structure, or grammar.

Topic 1: Tell the story of your illness – from when you were diagnosed to now

- What do you think of as the 'key' or 'most important' moments in your illness journey?
- Have there been any particularly difficult moments? How did you feel at the time? How do you feel about that moment now?
- Taking a step back and looking at the bigger picture, try to write a few sentences expressing understanding for the way you feel about those difficult times, and consider how this is something that anyone may feel.

Topic 2: Share your thoughts and feelings about being referred to the hospice

- What does the word *hospice* make you think of? How did you feel when you were first referred? How do you feel now? Have your feelings about receiving care from the hospice changed over time? Are there any good points? Any bad points?
- Taking a step back, can you understand why you may have felt or feel this way about hospice care?
- How do you think your feelings compare to other people who also receive hospice care? What would you say to somebody who is being referred to a hospice?

Topic 3: Tell the story of one or more of your happiest memories

- Think of the most wonderful experiences in your life. What have been some of the happiest or most significant moments for you? This could be a big event, a beautiful scene, falling in love or listening to music. Anything that brought you joy or felt meaningful to you. Describe those moments. How did you feel then? How does it make you feel thinking about them?
- Why do you think these moments feel so significant for you?

Remember to finish each session by writing a few sentences expressing warmth and kindness for yourself and your feelings.

Expression Session topics

- Follow the general instructions in the yellow box below for each Expression Session.
- The topics for each of the three Expression Sessions are described on the next page. Feel free to change the order or repeat a session of your choice if you wish. These topics are just a guide.
- At the beginning of each session, set your timer and try to write in response to the suggested topic for at least 20 minutes. If you become too tired to continue, feel free to pause and pick back up again when you feel able. If at the end of 20 minutes you feel you have more to write, set the timer for another 10 minutes and keep going until you feel ready to stop. We would not recommend you write for longer than 30 minutes in total though.

General Instructions

Keep these in mind throughout each Expression Session

- As you are writing, try to remember and describe the thoughts and emotions you felt during each experience.
- Some of the moments you describe might be painful or difficult to remember. As you describe these experiences, try to write from the perspective of someone who is accepting and understanding of your emotions, and consider how other people may feel the same way when in similar situations. Try to write with kindness and concern for yourself in mind. It might help to think about what you would say to a friend if they were in your shoes, or what a friend might say to you.
- At the end of each session, try to finish by writing a few sentences focussing on expressing warmth and kindness for yourself and your feelings.

Don't worry about spelling, sentence structure, or grammar.

Topic 1: Tell the story of your illness – from when you were diagnosed to now

- What do you think of as the 'key' or 'most important' moments in your illness journey?
- Have there been any particularly difficult moments? How did you feel at the time? How do you feel about that moment now?
- Taking a step back and looking at the bigger picture, try to write a few sentences expressing understanding for the way you feel about those difficult times, and consider how this is something that anyone may feel.

Topic 2: Share your thoughts and feelings about being referred to the hospice

- What does the word *hospice* make you think of? How did you feel when you were first referred? How do you feel now? Have your feelings about receiving care from the hospice changed over time? Are there any good points? Any bad points?
- Taking a step back, can you understand why you may have felt or feel this way about hospice care?
- How do you think your feelings compare to other people who also receive hospice care? What would you say to somebody who is being referred to a hospice?

Topic 3: Tell the story of one or more of your happiest memories

- Think of the most wonderful experiences in your life. What have been some of the happiest or most significant moments for you? This could be a big event, a beautiful scene, falling in love or listening to music. Anything that brought you joy or felt meaningful to you. Describe those moments. How did you feel then? How does it make you feel thinking about them?
- Why do you think these moments feel so significant for you?

Remember to finish each session by writing a few sentences expressing warmth and kindness for yourself and your feelings.

Good Reporting of A Mixed Methods Study (GRAMMS) checklist[1]

Guideline	Section: page
Describe the justification for using a mixed methods approach to the research question	Methods and analysis (Design): page 5
Describe the design in terms of the purpose, priority and sequence of methods	Methods and analysis (Data collection): page 9 - 13
Describe each method in terms of sampling, data collection and analysis	Methods and analysis (Data collection and analysis): page 9 - 15
Describe where integration has occurred, how it has occurred and who has participated in it	Methods and analysis (Data analysis – primary objective): page 13-14
Describe any limitation of one method associated with the present of the other method	Methods and analysis (Design): page 5
Describe any insights gained from mixing or integrating methods	Tbc – protocol therefore results not reported (see Methods [design section] for justification / aims)

1. O'Cathain A, Murphy E, Nicholl J. The quality of mixed methods studies in health services research. *J Health Serv Res Policy.* 2008;13: 92-98.

For peer review only

BMJ Open

Let It Out (LIO) study: protocol for a mixed-methods study to optimise the design and assess the feasibility of an online emotional disclosure-based intervention in UK hospices

Journal:	*BMJ Open*
Manuscript ID	bmjopen-2020-047135.R1
Article Type:	Protocol
Date Submitted by the Author:	12-Mar-2021
Complete List of Authors:	McInnerney, Daisy; UCL, Marie Curie Palliative Care Research Department, Division of Psychiatry<br>Candy, Bridget; UCL, Marie Curie Palliative Care Research Department, Division of Psychiatry<br>Stone, Patrick; UCL, Marie Curie Palliative Care Research Department, Division of Psychiatry<br>Kupeli, Nuriye; UCL, Marie Curie Palliative Care Research Department, Division of Psychiatry
**Primary Subject Heading**:	Palliative care
Secondary Subject Heading:	Research methods, Mental health
Keywords:	PALLIATIVE CARE, PSYCHIATRY, MENTAL HEALTH, STATISTICS & RESEARCH METHODS, QUALITATIVE RESEARCH

SCHOLARONE™
Manuscripts

I, the Submitting Author has the right to grant and does grant on behalf of all authors of the Work (as defined in the below author licence), an exclusive licence and/or a non-exclusive licence for contributions from authors who are: i) UK Crown employees; ii) where BMJ has agreed a CC-BY licence shall apply, and/or iii) in accordance with the terms applicable for US Federal Government officers or employees acting as part of their official duties; on a worldwide, perpetual, irrevocable, royalty-free basis to BMJ Publishing Group Ltd ("BMJ") its licensees and where the relevant Journal is co-owned by BMJ to the co-owners of the Journal, to publish the Work in this journal and any other BMJ products and to exploit all rights, as set out in our licence.

The Submitting Author accepts and understands that any supply made under these terms is made by BMJ to the Submitting Author unless you are acting as an employee on behalf of your employer or a postgraduate student of an affiliated institution which is paying any applicable article publishing charge ("APC") for Open Access articles. Where the Submitting Author wishes to make the Work available on an Open Access basis (and intends to pay the relevant APC), the terms of reuse of such Open Access shall be governed by a Creative Commons licence – details of these licences and which Creative Commons licence will apply to this Work are set out in our licence referred to above.

Other than as permitted in any relevant BMJ Author's Self Archiving Policies, I confirm this Work has not been accepted for publication elsewhere, is not being considered for publication elsewhere and does not duplicate material already published. I confirm all authors consent to publication of this Work and authorise the granting of this licence.

For peer review only

Let It Out (LIO) study: protocol for a mixed-methods study to optimise the design and assess the feasibility of an online emotional disclosure-based intervention in UK hospices

Authors:

Daisy McInnerney[1a], Bridget Candy[1], Patrick Stone[1], Nuriye Kupeli[1]

Affiliations:

[1] Marie Curie Palliative Care Research Department, Division of Psychiatry, University College London, London, UK

Corresponding author:

a. Wing B, Floor 6, Maple House, 149 Tottenham Court Road, London, W1T 7NF; daisy.mcinnerney.18@ucl.ac.uk

Word count: 3901

For peer review only

ABSTRACT

Introduction: The current COVID-19 pandemic has forced hospices to look for more ways to support people remotely, including psychological support. Emotional disclosure-based interventions hold potential as a way of providing support remotely. However, evidence of their efficacy in people with terminal illness is mixed. Reviews have highlighted this may be due to interventions not being tailored to the unique needs of this population. In response to this, we are developing Let It Out (LIO), an online, self-guided emotional disclosure-based intervention tailored for people living with terminal illness.

Aims: The primary objective of the study is to optimise the design of the LIO intervention. Secondary objectives include assessing its acceptability and feasibility; exploring potential impact on wellbeing; identifying potential adverse effects; and informing choice of outcome measures for potential future evaluation.

Methods and analysis: A single arm, mixed-methods, multi-site longitudinal study. Up to 40 people living with a terminal illness under the care of hospices in England and Scotland will receive the online LIO intervention. LIO consists of three, self-guided expression sessions over two weeks. The primary outcome measures are (1) a structured feedback form completed by participants after the final expression session; and (2) semi-structured interviews and focus groups with ≤15 patient participants, ≤30 UK hospice staff and ≤15 informal carers. These quantitative and qualitative data will be triangulated via process evaluation to inform optimisation of the intervention design. Secondary outcome measures include validated measures of physical and psychological health collected at baseline and after the final expression session (immediately, one, four and eight weeks after); and data on recruitment; retention; and fidelity.

Ethics and dissemination: The study is approved by the University College London Research Ethics Committee (reference: 15281/002). The findings will be shared through peer-reviewed scientific journals and conferences, and traditional, online and social media platforms.

KEYWORDS

PALLIATIVE CARE, PSYCHIATRY, MENTAL HEALTH, STATISTICS & RESEARCH METHODS, QUALITATIVE RESEARCH

For peer review only - http://bmjopen.bmj.com/site/about/guidelines.xhtml

ARTICLE SUMMARY

Strengths and limitations of this study

- The LIO intervention has been developed based on evidence from a scoping review of previous research, and refined drawing on insights from testing it in a slightly different form in another population and feedback from key stakeholders.
- The acceptability and feasibility of the intervention will be explored using a rich, mixed-methods approach, using process evaluation to triangulate quantitative recruitment, retention and acceptability data with qualitative written and interview feedback.
- Recruitment may be restricted by the relatively short recruitment period and potential limitations on hospice staff capacity (due to COVID-19 related pressures on hospice teams).
- The study is taking place during a time of transition and ongoing change in methods of care delivery, necessitated by the evolving COVID-19 pandemic that may restrict generalisability of the findings outside of this context.
- This is a single-arm study not designed to formally evaluate efficacy of the intervention.

For peer review only - http://bmjopen.bmj.com/site/about/guidelines.xhtml

INTRODUCTION

People living with terminal illness carry a unique emotional burden and can experience significant distress.(1–4) Psychological support is a key part of the holistic care provided by hospices and other palliative care services.(5–8) But evidence suggests psychological service provision at the end-of-life is currently inadequate, and services are often under-resourced and over-burdened.(9–11) Since the start of the COVID-19 pandemic in early 2020, hospice funding and capacity have been placed under even more threat.(12) Hospices are increasingly turning to telehealth approaches to deliver care to patients.(13) At present this is necessary to help minimise face-to-face contact in line with recommended social distancing practices that are particularly crucial for this vulnerable population.(14) However, delivery of care using such methods is potentially cost- and time-effective, and if found to be beneficial, may endure beyond the resolution of the pandemic to help broaden access to palliative care.

Emotional disclosure (ED)-based therapies, such as expressive writing or talking, are easy to implement as a means of support, with minimal requirement for staff supervision and potential to be delivered online.(15,16) The traditional expressive writing protocol is based on the principle that expressing feelings about personal, traumatic experiences can provide comfort.(17) Indeed, there is evidence that such interventions can provide psychological and physical benefits in healthy populations.(18) Expressive writing has also been adapted, to include, for example disclosing about positive events,(15,19,20) writing or talking about stress from a compassionate stance (21–23) or writing about future goals.(24,25) Writing from a compassionate stance has been shown to reduce negative affect (22,26) and improve mood.(27) However, evidence of benefit of ED-based interventions in palliative populations is more mixed.(28,29) The mixed results may be because there has been a lack of research into developing an ED-based intervention tailored to the specific needs of this population.(28) The underlying causal mechanisms, and thus the outcome measures that may be most suitable to capture any potential benefit of such an intervention in this population, also remain unclear.

Based on this gap in the literature, our group conducted a scoping review of ED-based interventions in people with terminal illness.(29) The review aimed to identify potentially effective characteristics of interventions for this population to inform the development of a tailored ED-based intervention. The review identified 25 relevant primary studies, but found that quantitative evidence was not available to determine which, if any, characteristics may be most effective.(*McInnerney et al., 2020, manuscript under review*) However, the qualitative analysis of authors' experiences of testing the interventions highlighted a number of themes that may be important to consider when developing an ED-based intervention for people with terminal illness. These include flexibility of when, where and how to complete the intervention, clarity of instructions and provision of a safe environment for disclosure.

For peer review only

For peer review only - http://bmjopen.bmj.com/site/about/guidelines.xhtml

Building on the results of this scoping review, we developed an ED-based intervention in collaboration with key stakeholders named Let It Out (LIO). We then refined its design by incorporating insights from a trial we ran of an adapted version of the intervention tested in the general population during the COVID19 pandemic,(30) and further stakeholder feedback. This study represents the next phase of the intervention development process.

Aims and objectives

The primary objective of the study is to optimise the design of an ED-based intervention (LIO) tailored for people receiving palliative care from a UK hospice. The secondary objectives are to:

1. Assess whether the intervention and study procedures are acceptable and feasible in UK hospice services.
2. Explore the potential impact of the intervention on psychological and physical wellbeing, and identify any potential adverse effects.
3. Inform the choice of outcome measures and study design for possible future large-scale intervention evaluation.
4. Explore the causal mechanisms underlying potential intervention efficacy.

An exploratory objective is to explore the core concerns of people following referral to hospice care. This is one of the topics covered in the intervention expression sessions; responses could potentially inform strategies to minimise or address those concerns.

METHODS AND ANALYSIS

Design

A pragmatic, single-arm, multi-site, mixed-methods longitudinal study. The study design has been developed in line with the latest Medical Research Council (MRC) guidelines,(31,32) which recommend an iterative intervention development and evaluation process. Mixed-methods, pragmatic study designs are recognised as an appropriate way for researchers to approach the development and evaluation of complex interventions in palliative care.(33) We are employing a convergent design to integrate quantitative measures of fidelity, feasibility, acceptability and possible impact, with semi-structured interviews and focus groups. In the qualitative aspect of the study, we will take a phenomenological approach to capture more nuanced feedback on the participants' experience of the intervention, including their views on its design, acceptability and any perceived effects.(33,34) The quantitative and qualitative data will be integrated through a process evaluation.

For peer review only - http://bmjopen.bmj.com/site/about/guidelines.xhtml

As the focus of the study is on applying pragmatic mixed-methods to optimise intervention design, this study will not formally evaluate nor test *a priori* hypotheses regarding its efficacy or report p-values.

The study is approved by the University College London Research Ethics Committee (reference: 15281/002).

Setting

The study will take place in six UK hospice services across England and Scotland. A list of participating sites is available from the authors.

Participants

The study will be conducted in three population groups recruited from participating hospices:

Group 1: Up to 40 adults living with terminal illness receiving palliative care from a UK hospice (on an inpatient, day care, outpatient or community basis). A sample size of 40 is recognised by the National Institute of Health Research as being appropriate for intervention feasibility and pilot work that is not designed to evaluate efficacy.(35,36)

Group 1 exclusion criteria

- Not expected to survive for longer than ten weeks (based on clinical judgement).
- Lack of capacity to give informed consent or may be expected to lose capacity to give informed consent within ten weeks (based on clinical judgement).
- Known diagnosis or history of severe depression, suicidal tendencies, psychotic symptoms, severe mood disorders, mania, schizophrenia, psychopathic/borderline personality disorder, severe and episodic (Type 1) bipolar (affective) disorder (that is not well controlled).
- Not able to understand and communicate in English.

Group 2: Up to 15 informal (adult friend or family) carers of Group 1 participants.

Group 3: Up to 30 paid or voluntary hospice staff involved in organisation, delivery or implementation of psychological and/or emotional support.

Recruitment

Group 1

We will employ convenience sampling. This is a pragmatic decision based on difficulties recruiting participants during the COVID-19 pandemic; the target numbers for recruited participants are in place to manage the scope

For peer review only - http://bmjopen.bmj.com/site/about/guidelines.xhtml

of the research. We will assess recruitment rate as part of the study's feasibility assessment. The recruitment window has been selected based on research team capacity and funding availability.

Clinical and research staff at hospice sites will identify patients who may be eligible to participate between October 2020 and January 2021. They will make initial contact, explain the study and if the participant is interested, provide them with an invitation letter, email or link to the study website. The study website hosts the participant information sheet and a link to an online reply form. Participants can indicate on the reply form if they would or would not like to take part.

If participants select yes on the reply form, they can progress to the online informed consent and advance consent form, which asks what participants would like to happen to their data if they lose capacity over the course of their participation in the study. If they select no, they have the option to provide their reasons for not taking part. There is also an option to request further information from the research team before making a decision. If participants select this option, they are prompted to provide their contact details and a member of the research team will phone or email them to discuss the study further. If, having discussed the study, participants choose to take part, they can progress to the online informed consent and advance consent forms. The research team will inform the relevant hospice when a participant from their service consents to take part, so it can be recorded on their patient notes. The participant will be reminded of their right to withdraw from the study at any time throughout the study in online materials and through direct communication with the research team.

In the initial phase of the study, all Group 1 participants will be invited to take part in a semi-structured interview. Depending on uptake, once a third (n=5) have been recruited to interview phase, we will assess the mix of participants in terms of demographic characteristics, to inform recruitment targets according to a purposive sampling framework.

Group 2 (informal carers) will be recruited through researcher contact with Group 1 participants. If their carer indicates they may be interested in taking part in an interview, the research team will arrange a time to discuss the study; if interested in taking part, they will be sent a link to the online participant information sheet and informed consent form.

For peer review only - http://bmjopen.bmj.com/site/about/guidelines.xhtml

Group 3 (staff and volunteers) will be recruited through liaison with clinical leads at each hospice site. The research team will approach eligible staff members to introduce the study, and if interested, provide them with a link to the online participant information sheet and informed consent form.

The study will be presented at hospice staff team meetings and internal newsletters to support recruitment.

The intervention

Development

The Let It Out (LIO) intervention has been developed in collaboration with a group of stakeholders, including psychologists, a psychiatrist, palliative care and hospice staff, palliative care specialist researchers and Patient and Public Involvement (PPI) representatives. The intervention was first developed based on the findings of a scoping review of related interventions conducted by the research team.(29) Based on the review findings, the LIO intervention is flexible in terms of when, where and how it can be completed; provides clear, structured instructions; and emphasises the importance of completing sessions in a safe, comfortable setting. During the COVID-19 pandemic, the research team adapted the intervention to test its effect on distress in the general population during the pandemic (the LIO-C study).(30) Based on feedback from participants in that study, adaptations were made to LIO to minimise any potential risk of causing short-term distress; namely, by incorporating clearer directions on how to express feelings with self-compassion.

Theoretical basis

The intervention development process has been guided by emotion regulation theory, which posits that the active component of ED-based interventions is the emotional arousal following expression of emotions.(37–39) This account suggests that one is able to develop acceptance of their thoughts and feelings through mastery, self-efficacy and control of their emotions. This account also sits within theories underlying positive psychology and self-compassion interventions,(19,40) which propose that one is able to gain a better understanding of the emotions associated with their experiences by learning how to more helpfully structure thoughts and feelings. We recognise that there is unlikely to be a single process underlying ED; indeed, several alternative processes have been proposed to explain ED including emotional inhibition, cognitive adaptation and exposure.(41–44) One aim of this study, in line with the MRC complex intervention development guidance,(32) is to explore the mechanisms through which LIO may be effective.

Outline

LIO is a self-guided intervention designed to enable people living with terminal illness to self-compassionately express their feelings in a way that may help to bring them comfort. In this study, participants will be sent a

For peer review only - http://bmjopen.bmj.com/site/about/guidelines.xhtml

link to online intervention instructions, as well as a PDF intervention guide that can be downloaded from the online survey platform. The guide provides instructions for participants to work through the study and intervention. Over two weeks, participants are asked to express their feelings in a self-compassionate way in response to prompts in three separate, 20-30 minute 'expression sessions'. Figure 1 presents the general instructions given to participants to use across each session and the prompts for each session.

Figure 1. **General instructions and session specific prompts as part of LIO guide (written version)**

Participants are advised to complete sessions in a comfortable place, and, if possible, in private (although it is recognised this may not be feasible or wanted for all participants). Participants can handwrite, type or audio-record (using their personal mobile phone or laptop) their response to the prompts. There are two guides: one for those choosing to write, the other for those choosing to audio-record. The full guides are available on reasonable request from co-authors.

To make the intervention as accessible as possible, it is acceptable for participants to have support from a carer to help complete the expression sessions, and the associated questionnaires. This help can be delivered in any way that works best for the participant. The guide and website include signposts to other helpful resources that can support mental wellbeing. The guide also provides a space for participants to plan when and where they will complete each session, based on the theory of action-planning to help bridge the intention-behaviour gap.(45)

Data collection

Overview – Group 1

Table 1 provides a detailed description of the participant timeline and outcome measures for Group 1 participants.

Expression sessions – Group 1

Over the two week intervention period, participants will type, handwrite or audio-record (format chosen by the participant) their response to the three prompts. Handwritten and audio-recorded responses will be transcribed for analysis. It is not mandatory for participants to share their expression session responses if they would prefer to keep them private. Methods for data collection and transfer are summarised in Table 2. Researchers will contact each participant by phone or email (participant preference) at the end of the first and second week of the intervention to check wellbeing. If during contact with the participant the research team suspects they may no longer have capacity for informed consent, or is experiencing severe distress, the hospice clinical team will be informed and capacity formally assessed. If the participant is deemed no longer

For peer review only - http://bmjopen.bmj.com/site/about/guidelines.xhtml

eligible to take part in the study, they will be withdrawn and the directives from their advance consent form followed.

Self-report measures – Group 1

All self-report data is collected online using the REDCap online survey platform (46,47). Group 1 participants will complete a demographics questionnaire at baseline (prior to receiving the intervention). They will also complete a series of health-related questionnaires at baseline and one, four, and eight weeks after the final intervention session (secondary outcome measures). Group 1 participants will also be prompted to log in to the platform during or after each expression session to indicate they have completed the session, and (if they wish) to share their response with the research team. Directly after each expression session they will also be prompted to complete a short self-report questionnaire to monitor their mood, and perception of how meaningful and personal the session was (secondary outcome measures). After the final expression session, participants will also complete a feedback form (primary outcome measure).

Semi-structured individual interviews (primary outcome measure) – Group 1

Semi-structured interviews (n≤15) will be conducted within 8 weeks of the participant's final expression session. Interviews will be conducted by DM, following a topic guide to explore participants' experience of the intervention and questionnaires, including whether they experienced any negative feelings during or after each expression, whether they found the intervention helpful, and the appropriateness and relatability of the expression session prompts, expanding on the topics covered in the feedback form.

Interviews will take place virtually and will be audio-recorded using Microsoft Teams, to minimise face-to-face interactions in line with government COVID-19 guidance. Recordings will be transcribed verbatim for analysis; transcripts will be pseudonymised and personally identifiable information redacted from the transcript.

For peer review only

For peer review only - http://bmjopen.bmj.com/site/about/guidelines.xhtml

Table 1. Group 1 participant timeline, outcome measures and analysis approach

Measure	Timepoints administered	Description	Data type	Analysis approach	Relevant research objective*
Demographics	Baseline	Approximate date of hospice referral, age, gender, ethnicity, marital status, religious background, level of education, type of care (eg, inpatient, outpatient, day care), diagnosis, mental health history and COVID19 status.	Quantitative	Descriptive statistics	1. Feasibility and acceptability
Expression Sessions	3 sessions over 2-week intervention period	Typed, handwritten or audio-recorded responses to three expression sessions that make up the LIO intervention.	Quantitative and Qualitative	LIWC and BIR<br><br>Inductive thematic analysis (Session 2)	1. Feasibility and acceptability<br><br>4. Underlying mechanisms<br><br>Exploratory objective (Session 2)
Health-related outcome measures (see below for details):	Baseline, immediately after the final expression, and, 1-week, 4-weeks and 8-weeks after the final expression	*See below for details*	Quantitative	Descriptive statistics and descriptive pre-post analysis	1. Feasibility and acceptability<br><br>2. Impact on wellbeing<br><br>3. Outcome measure selection<br><br>4. Underlying mechanisms
Integrated Palliative Care Outcome Scale		An updated, brief (10 questions, 17 items) version of the POS instrument. IPOS was developed specifically to			

For peer review only - http://bmjopen.bmj.com/site/about/guidelines.xhtml

(IPOS) – patient version (48)	capture a range of the most important concerns of people living with palliative-stage disease, covering symptoms, information needs, practical concerns, anxiety or low mood, family anxieties and overall feeling of being at peace. Items in question 2 to 9 are scored from 0 to 4, and overall scores can range from 0 to 68. Questions 1 and 10 are not scored.
Patient Health Questionnaire (PHQ) 9 (49)	A 9-item instrument developed to screen for major depressive disorder, and validated in palliative care populations (50). Items are scored 0 to 3, and it can be scored continuously or with validated cut-offs for mild (5–9), moderate (10–14), moderately severe (15–19) and severe depression (20–27) (51,52). The cut-off for a depression “case” is 10.
Generalised Anxiety Disorder (GAD) 7 (53)	A validated 7-item anxiety screening instrument that is used to screen for anxiety in palliative care settings. Items are scored 0 to 3, and total scores range from 0 to 21. Severity of anxiety symptoms can be classed as None (0-4), Mild (5-9), Moderate (10-14) or Severe (15-21). The cut-off score for a Generalised Anxiety Disorder “case” is 10.
Single-item Sleep Quality Scale (SQS) (54)	A short, pragmatic, tool for assessing sleep quality that has been validated in people with insomnia and depression, selected to reduce burden on participants from lengthier alternatives such as the Pittsburgh Sleep Quality Index

For peer review only - http://bmjopen.bmj.com/site/about/guidelines.xhtml

		(PSQI) (55). A single visual analogue scale asks respondents to rate their overall quality of sleep over the last seven days on a scale of 0 to 10. Scores are used to categorise sleep quality as: Terrible (0), Poor (1-3), Fair (4-6), Good (7-9) and Excellent (10).			
Uptake of existing mental health services		A three-item form developed by our research team specifically for this study asking participants if they have (1) ever sought help from a healthcare professional for their mental health; (2) sought help from a healthcare professional for their mental health over the past two weeks; and (3) any plans to seek help from a healthcare professional for their mental health in the future. Response options are 'Yes', 'No', or 'Prefer not to say'.			
Meaningfulness / personal / mood ratings	Immediately after each expression session	A 3-item, 7-point Likert scale based on a measure used in a study testing a similar intervention (56), measuring how personal and meaningful participants' felt their expression session was (not very personal/not very meaningful (-3) to (+3) very personal/very meaningful). The last item will be a mood check where participants are asked to rate how they feel on a 7-point scale (very sad (-3) to (+3) very happy).	Quantitative	Descriptive statistics	1. Feasibility and acceptability<br>2. Impact on wellbeing<br>3. Outcome measure selection<br>4. Underlying mechanisms

For peer review only

For peer review only - http://bmjopen.bmj.com/site/about/guidelines.xhtml

Feedback form	Immediately after final expression session	Participants will be asked to complete an online feedback form exploring their experience of completing the intervention, if they needed any assistance from a carer and the nature of that assistance, and their fidelity to the protocol. The feedback form questions were informed by work on ethical research in vulnerable populations (57) and The Theoretical Framework of Acceptability for healthcare system interventions (58).	Quantitative and qualitative	Descriptive statistics and combined deductive and inductive thematic analysis	1. Feasibility and acceptability<br>2. Impact on wellbeing<br>3. Outcome measure selection<br>4. Underlying mechanisms
Expression Sessions	3 sessions over 2-week intervention period	Typed, handwritten or audio-recorded responses to three expression sessions that make up the LIO intervention	Quantitative and Qualitative	LIWC and BIR<br>Inductive thematic analysis (Session 2)	1. Feasibility and acceptability<br>4. Underlying mechanisms<br>Exploratory objective (Session 2)
Semi-structured interview	Within 8 weeks of final expression session	Exploring participants' experience of the intervention and questionnaires, including whether they experienced any negative feelings during or after each expression, whether they found the intervention helpful, and the appropriateness and relatability of the expression session prompts, expanding on the topics covered in the feedback form	Qualitative	Combined deductive and inductive thematic analysis	1. Feasibility and acceptability<br>2. Impact on wellbeing<br>3. Outcome measure selection<br>4. Underlying mechanisms

256

For peer review only - http://bmjopen.bmj.com/site/about/guidelines.xhtml

Abbreviations: BIR, Blinded Independent Review; GAD-7, Generalised Anxiety Disorder-7; IPOS, Integrated Palliative Care Outcome Scale; LIWC, Linguistic Inquiry Word Count; PHQ-9, Patient Health Questionnaire-9; SQS, Single-item Sleep Quality Scale

*All measures will contribute to the primary objective: optimisation of the intervention as informed by a process evaluation (see Table 3).

For peer review only

For peer review only - http://bmjopen.bmj.com/site/about/guidelines.xhtml

Table 2. Methods of expression session data collection and transfer for Group 1 participants

Method of disclosure	Data collection	Method for transfer to research team
Typed into platform	Participant types directly into text box on online survey platform (REDCap via Data Safe Haven).	Research team have access to data entered onto REDCap via Data Safe Haven.
Typed on device	Participant types into word processor on own device.	Participant saves file on own device and uploads it to the REDCap via Data Safe Haven survey platform.
Hand-written	Participant handwrites responses on own paper.	Participant takes photo of handwritten responses using mobile phone camera and sends via WhatsApp to research team* (end-to-end encryption). Photos transferred to Data Safe Haven and deleted from phone.
Audio-recorded on smart phone	Participant records themselves using either inbuilt recording / notes app on their phone or the Rev app (free to download).	Participant sends audio-recording via WhatsApp to research team* (end-to-end encryption). Audio-recording transferred to Data Safe Haven and deleted from phone.
Audio-recorded – on laptop/computer	Participant records themselves using free Audacity software.	Participant saves file on own device and uploads it to the REDCap via Data Safe Haven survey platform.

*The research team will have a dedicated study phone that a member of the research team keeps only on their person during working hours (9am – 5pm Monday – Friday) and is kept in a secure locked cabinet outside of those hours. The phone will be checked once a day for recordings or photos received, at which point the recording will be transferred to the dedicated project file on Data Safe Haven and deleted from the phone.

For peer review only

For peer review only - http://bmjopen.bmj.com/site/about/guidelines.xhtml

Overview - Group 2 and 3 (primary outcome measures)

Semi-structured interviews will also be conducted with ≤15 informal carers of Group 1 participants after they have completed their final expression session. Focus groups or semi-structured interviews (depending on practicalities of arranging a suitable time) with ≤30 hospice staff and volunteers will also be conducted. As with Group 1, interviews and focus groups will be conducted by DM, and will follow topic guides designed to explore participants' views on the design and content of LIO; if and how it may need to be adapted; potential risks of harm or benefit; where LIO might fit into current pathways of care; and what help may be needed to support its implementation. All Group 2 and 3 participants will complete an online demographics questionnaire prior to taking part in the interview/focus group.

As with Group 1, interviews and focus groups will take place virtually and will be audio-recorded using Microsoft Teams. Recordings will be transcribed verbatim, pseudonymised and personally identifiable information redacted from the transcript for analysis.

Data analysis

Primary objective: process evaluation and intervention proposal

Using the qualitative and quantitative data collected from Groups 1, 2 and 3 (and in combination with evidence that has informed this research protocol from prior work (reviews and a trial) conducted by the research team), a triangulation approach will be applied drawing on process evaluation methodology.(34,59) Process evaluation is recognised as a valuable, pragmatic method to optimise intervention design and inform evaluation during feasibility testing phases.(34) Through the process evaluation, we aim to develop a preliminary theoretical model to inform understanding of potential causal mechanisms of LIO and any contextual and implementation factors that may be associated with any potential variation in outcomes. Insights from the process evaluation will inform the final intervention proposal. **Table 3** describes the framework that will be used to guide the integration of mixed-methods data for the process evaluation. We developed the framework based on the MRC guidance on conducting process evaluations (34).

Analysis will be led by DM in iterative consultation with the research team and advisory group. Codes, themes, descriptive statistics, data integration and conclusions generated by DM will be checked at key stages throughout the project by the research team. This will help minimise any risk of a positivity bias arising from DM's close connection to the intervention being developed, and help validate the integrity of DM's interpretation of the data. Thematic analysis will be carried out using QSR International Nvivo 11.4 software (60). Quantitative responses will be summarised using SPSS Version 24 (61).

For peer review only - http://bmjopen.bmj.com/site/about/guidelines.xhtml

Secondary objectives

Feasibility and acceptability

Due to the preliminary nature of this study, no pre-specified criteria have been defined to establish acceptability of the LIO intervention, or the feasibility of the study procedures. The following measures of feasibility and acceptability will be analysed:

A. Recruitment and retention

i. Descriptive statistics of recruited participant numbers and demographics.
ii. Percentage of participants recruited completing each data collection time-point.

B. Adherence

i. Percentage of participants completing all three expression sessions (a) within 20-30 minute time frame and (b) in a private space.
ii. Blinded independent assessor review of texts to determine which prompt was being responded to.
iii. Linguistic Inquiry Word Count (LIWC) analysis of expression session texts/transcripts (62) to analyse percentages of emotion, self and prompt-related words. LIWC is a computer programme that calculates word count, and percentages of words used that reflect different emotions, thinking styles and concerns.

C. Acceptability

i. Descriptive quantitative analysis of responses to the feedback form.
ii. Combined deductive and inductive qualitative thematic analysis of free-text responses to feedback form and interviews with patient, carer and staff participants. (63) Data will be coded to the seven constructs of The Theoretical Framework of Acceptability (TFA) for healthcare system interventions (deductive component).(58) Within each construct, further codes will be developed from the raw data (inductive component). DM will lead the application and development of codes. These will then be checked and adjusted by the research team (intercoder review and code testing), and data re-coded. This process will be repeated iteratively until code/theme saturation is reached.(64)

For peer review only - http://bmjopen.bmj.com/site/about/guidelines.xhtml

Impact on psychological and physical wellbeing

A. Inductive thematic analysis of interviews and feedback from free-text responses from patients, carer and staff participants. DM will develop codes from the raw data, to be checked and adjusted by the research team; data will be re-coded, checked and adjusted until code saturation is reached.
B. Descriptive pre-post analysis of changes in outcome measures (IPOS, HADS, GAD7, SQS, uptake on mental health services): mean and standard deviation of scores at each timepoint.
C. Descriptive summary of number of adverse effects reported by clinical team and participants.

Choice of outcome measure: inferences of potentially suitable outcome measures will be based on results from descriptive pre-post analysis of outcome measures, and qualitative analysis of interviews and feedback from free-text responses from patients, carers and staff participants regarding reported impact of the intervention.

Potential underlying mechanisms: responses to expression session prompts will be analysed quantitatively using LIWC software (62) and triangulated with indicators of intervention impact on wellbeing to inform development of a preliminary theoretical model.

Core concerns on referral to hospice: responses to expression session prompts will be analysed using inductive thematic analysis. DM will develop codes from the raw data, to be checked and adjusted by the research team; data will be re-coded, checked and adjusted until code saturation is reached.

For peer review only

For peer review only - http://bmjopen.bmj.com/site/about/guidelines.xhtml

Table 3. Framework guiding analysis and integration of mixed-methods data for process evaluation

	Research questions	Data source	Data analysis
Implementation	**Coverage and reach (retention):**<br>• What percentage of Group 1 participants completed the intervention?<br>• What are the demographics of participants who took part in the study and completed the intervention?<br>• What were the reasons given for non-participation?	Recruitment / data collection records<br>Demographics questionnaire<br>Researcher reflective notes and informal feedback from research sites; withdrawal questionnaire	Descriptive statistics<br>Inductive thematic analysis
	**Fidelity, frequency and duration (adherence):**<br>• To what extent was the intervention implemented as planned?<br>○ What percentage of participants completed all three expression sessions, within the 20-30 minute timeframe, in a private space?<br>○ What percentage of expression sessions were assigned to the correct prompt by a blinded assessor?<br>○ To what extent do expression session transcripts use Emotion and Positive Emotion words?	Recruitment / data collection records<br>Group 1 feedback form<br>Interviews and focus groups with Group 1, 2 and 3<br>Expression session texts	Descriptive statistics<br>Blinded Assessor Review<br>LIWC analysis
	**Acceptability:**<br>• How acceptable is the intervention for people living with terminal illness receiving care from hospices, their informal carers and hospice staff?	Group 1 feedback form<br>Interviews and focus groups with Group 1, 2 and 3	Descriptive analysis and inductive thematic analysis coded to concepts in the TFA

For peer review only - http://bmjopen.bmj.com/site/about/guidelines.xhtml

	• What factors affect its acceptability?		
**Impact and mechanisms**	**Impact:**<br>• What are the perceived benefits of the intervention from the perspective of people living with terminal illness, their informal carers and hospice staff?<br>• Are there any changes in scores of psychological and physical health measures before and after (immediately, 1-, 4 and 8-weeks post intervention)?	Group 1 feedback form<br>Interviews and focus groups with Group 1, 2 and 3<br>Physical and psychological health-related outcome measures and mood checks	Impact:<br>Inductive thematic analysis<br>Descriptive pre-post statistics
	**Mechanisms of impact:**<br>• What cognitive processes are taking place during each Expression Session?<br>• Are there any patterns in the characteristics of participants who perceive benefits?	Expression Session texts<br>Demographic questionnaires	LIWC<br>Descriptive statistics
**Contextual factors**	What factors at political, economic, organisational and individual level affected, or might be expected to affect, the implementation and/or impact?<br>What options are there to minimise any barriers to successful implementation?	Group 1 feedback form<br>Interviews and focus groups with Group 1, 2 and 3<br>Researcher reflective notes and informal feedback from research sites; withdrawal questionnaire	Inductive thematic analysis

Abbreviations: LIWC, Linguistic Inquiry Word Count; TFA, The Theoretical Framework of Acceptability

For peer review only - http://bmjopen.bmj.com/site/about/guidelines.xhtml

Patient and Public Involvement (PPI)

The funding application for this programme of work was reviewed by Mr Peter Buckle, a Marie Curie PPI representative. Peter is also a member of the advisory group overseeing the conduct of the PhD studentship of which this study is a part. Peter, along with a second PPI representative (Dori-Anne Finlay) have reviewed the study and intervention design. Peter and Dori-Anne will be consulted at key stages of the research. Furthermore, the study as a whole has been designed based on the principles of co-design; the data from participants will be used to inform the design of the final intervention and potential future research.

Data management

All survey-based data (demographics and health-related questionnaires) will be collected using REDCap (46,47) via University College London Data Safe Haven. The Data Safe Haven has been certified to the ISO27001 information security standard and conforms to NHS Digital's Information Governance Toolkit. Built using a walled garden approach, the data is stored, processed and managed within the security of the system, avoiding the complexity of assured endpoint encryption. A file transfer mechanism enables information to be transferred into the walled garden simply and securely. A data management plan has been registered and approved by the University College London Data Protection Office outlining methods to maintain participant privacy and data integrity and confidentiality. At the end of the project, anonymised data will be uploaded to open-access data repository ReShare.(65)

ETHICS AND DISSEMINATION

Confidentiality

All participant data will be collected via the secure REDCap via Data Safe Haven platform,(46,47) or Microsoft Teams (interviews/focus groups), except audio-recordings or photographs of expression sessions, which will be sent directly to a dedicated study telephone via WhatsApp secured by end-to-end encryption. The phone will be carried by a member of the research team or stored in a locked drawer. Only members of the research team will have access to participant data. Consent and data collection forms will be securely stored in the University College London Data Safe Haven. During analysis, data will be pseudonymised using a unique ID that could not be linked to a participant's identity by anyone outside of the research team. Data will be anonymised 3-months after participants' involvement in the study ends.

For peer review only

Serious Adverse Events (SAE)

Any SAEs attributed to a person's participation in the study will be recorded on the online study platform, and in the participant's medical records. SAE forms will be provided by the chief investigator to the sponsor within five days of becoming aware of the event.

Dissemination

The findings of this study will be disseminated through peer-reviewed scientific journals, conferences, and traditional, online and social media.

For peer review only

ACKNOWLEDGEMENTS

The LIO study team are grateful to the group of advisors who have provided invaluable feedback on the design of the LIO intervention and study design to date: Peter Buckle, Jason Davidson, Dori-Anne Finlay, Dr Syd Hiskey, Dr Joana Johnson, Prof. Marc Serfaty, Dr Nick Troop. We are also grateful to the research and clinical leads and supporting staff at participating sites for their feedback on the intervention and study design, and support through the research governance process, particularly: Dr Anne Finucane, Dr Emma Carduff, Dr John MacArtney, Dr Amara Nwosu, Dr Adrian Tookman, Gail Holloway, Rachel Perry. Thank you also to Dr Sabine Best and Dr Briony Hudson for support in facilitating multi-site research across Marie Curie hospice sites.

AUTHOR CONTRIBUTIONS

The study and intervention design was led by DM. NK, BC and PS have collaborated at all stages on study and intervention development. NK is the Chief Investigator, based at University College London. DM is the PhD student managing the project. DM led the writing of this manuscript. All authors contributed to and approved the final draft.

FUNDING

This work was supported by Marie Curie (MCCC-FCH-18-U) and Economic and Social Research Council collaborative funding (ES/P000592/1). This work was also supported by core funding by Marie Curie (grant number: MCCCFPO-16-U) and an Alzheimer's Society (AS) funded fellowship awarded to NK (grant number: 399 AS-JF-17b-016).

COMPETING INTERESTS STATEMENT

None declared

REFERENCES

1. Galfin JM, Watkins ER. Construal level, rumination, and psychological distress in palliative care. Psychooncology. 2012 Jun;21(6):680–3.

2. Kolva E, Rosenfeld B, Pessin H, Breitbart W, Brescia R. Anxiety in Terminally Ill Cancer Patients. J Pain Symptom Manage. 2011 Nov;42(5):691–701.

3. Okuyama T, Endo C, Seto T, Kato M, Seki N, Akechi T, et al. Cancer patients' reluctance to discuss psychological distress with their physicians was not associated with underrecognition of depression by physicians: a preliminary study. Palliat Support Care. 2009 Jun;7(2):229–33.

4. Wilson KG, Chochinov HM, Graham Skirko M, Allard P, Chary S, Gagnon PR, et al. Depression and Anxiety Disorders in Palliative Cancer Care. J Pain Symptom Manage. 2007 Feb;33(2):118–29.

5. British Psychological Society. Psychological best practice in inpatient services for older people. 2017.

6. Cherny NI, on behalf of the ESMO Guidelines Working Group. ESMO Clinical Practice Guidelines for the management of refractory symptoms at the end of life and the use of palliative sedation. Ann Oncol. 2014 Sep 1;25(suppl 3):iii143–52.

7. Connor SR, Sepulveda Bermedo MC, Worldwide Palliative Care Alliance, World Health Organization. Global atlas of palliative care at the end of life. 2014.

8. Kalus C, Beloff H, Brennan J, McWilliams E, Payne S, Royan L, et al. The role of psychology in end of life care. The Professional Board of the British Psychological Society.; 2008.

9. Atkin N, Vickerstaff V, Candy B. 'Worried to death': the assessment and management of anxiety in patients with advanced life-limiting disease, a national survey of palliative medicine physicians. BMC Palliat Care [Internet]. 2017 Dec [cited 2019 Jan 9];16(1). Available from: https://bmcpalliatcare.biomedcentral.com/articles/10.1186/s12904-017-0245-5

10. Hospice UK. Impact of cost pressures - findings April 2019. 2019.

11. Russell C, Fountain A. Role of clinical psychology in UK hospices. BMJ Support Palliat Care. 2018 Oct 3;bmjspcare-2018-001594.

12. NHS. Funding arrangements for bed and community care capacity in the hospice sector [Internet]. 2020 [cited 2020 Jun 19]. Available from: https://www.england.nhs.uk/coronavirus/wp-content/uploads/sites/52/2020/04/CO365-grant-funding-arrangements-for-the-hospice-sector.pdf

13. Tavabie S, Ball K. Perspectives on dying from COVID-19: Waiting for the storm [Internet]. BMJ Supportive & Palliative Care. 2020 [cited 2020 Jun 17]. Available from: https://blogs.bmj.com/spcare/2020/03/26/perspectives-on-dying-from-covid-19-waiting-for-the-storm/

14. Public Health England. Guidance on shielding and protecting people who are clinically extremely vulnerable from COVID-19 [Internet]. GOV.UK. 2020 [cited 2020 Jun 19]. Available from:

For peer review only

For peer review only - http://bmjopen.bmj.com/site/about/guidelines.xhtml

https://www.gov.uk/government/publications/guidance-on-shielding-and-protecting-extremely-vulnerable-persons-from-covid-19/guidance-on-shielding-and-protecting-extremely-vulnerable-persons-from-covid-19

15. Baikie KA, Geerligs L, Wilhelm K. Expressive writing and positive writing for participants with mood disorders: An online randomized controlled trial. J Affect Disord. 2012;10.

16. Na S, S N, Pa F, Jw P, Rj O, Pp S, et al. Randomized Controlled Trial of Online Expressive Writing to Address Readjustment Difficulties Among U.S. Afghanistan and Iraq War Veterans [Internet]. Journal of traumatic stress. 2015 [cited 2020 Jun 19]. Available from: https://pubmed.ncbi.nlm.nih.gov/26467326/

17. Pennebaker JW, Beall SK. Confronting a Traumatic Event: Toward an Understanding of Inhibition and Disease. J Abnorm Psychol. 1986;95:274–81.

18. Frattaroli J. Experimental disclosure and its moderators: A meta-analysis. Psychol Bull. 2006;132(6):823–65.

19. Burton CM, King LA. The health benefits of writing about intensely positive experiences. J Res Personal. 2004 Apr;38(2):150–63.

20. Niles AN, Byrne Haltom KE, Lieberman MD, Hur C, Stanton AL. Writing content predicts benefit from written expressive disclosure: Evidence for repeated exposure and self-affirmation. Cogn Emot. 2016 Feb 17;30(2):258–74.

21. Imrie S, Troop NA. A pilot study on the effects and feasibility of compassion-focused expressive writing in Day Hospice patients. Palliat Support Care. 2012 Jun;10(2):115–22.

22. Przezdziecki A, Sherman KA. Modifying Affective and Cognitive Responses Regarding Body Image Difficulties in Breast Cancer Survivors Using a Self-Compassion-Based Writing Intervention. Mindfulness. 2016 Oct 1;7(5):1142–55.

23. Sherman KA, Przezdziecki A, Alcorso J, Kilby C, Elder E, Boyages J, et al. Reducing Body Image-Related Distress in Women With Breast Cancer Using a Structured Online Writing Exercise: Results From the My Changed Body Randomized Controlled Trial. J Clin Oncol. 2018 Jan 7;36(19):1930–40.

24. King LA. The Health Benefits of Writing about Life Goals. Pers Soc Psychol Bull. 2001 Jul;27(7):798–807.

25. Troop N, Chilcot J, Hutchings L, Varnaite G. Expressive writing, self-criticism, and self-reassurance. Psychol Psychother Theory Res Pract. 2013;86:374–86.

26. Leary MR, Tate EB, Adams CE, Batts Allen A, Hancock J. Self-compassion and reactions to unpleasant self-relevant events: The implications of treating oneself kindly. J Pers Soc Psychol. 2007;92(5):887–904.

27. Odou N, Brinker J. Exploring the Relationship between Rumination, Self-compassion, and Mood. Self Identity. 2014 Jul 4;13(4):449–59.

28. Kupeli N, Chatzithedorou G, Troop N, McInnerney D, Stone P, Candy B. Expressive writing as a therapeutic intervention for people with advanced disease: A systematic review. BMC Palliat Care.

For peer review only - http://bmjopen.bmj.com/site/about/guidelines.xhtml

29. McInnerney D, Kupeli N, Stone P, Anantapong K, Chan J, Candy B. Emotional disclosure as a therapeutic intervention in palliative care: a scoping review protocol. BMJ Open [Internet]. 2019 Aug 1 [cited 2019 Dec 16];9(8). Available from: https://bmjopen.bmj.com/content/9/8/e031046

30. McInnerney D, Kupeli N. Let It Out (LIO) and COVID19: Testing an Online Emotional Disclosure-based Intervention During the COVID19 Pandemic [Internet]. Clinicaltrials.gov. 2020 [cited 2020 Nov 5]. Available from: https://clinicaltrials.gov/ct2/show/NCT04386668

31. Craig P, Dieppe P, Macintyre S, Michie S, Nazareth I, Petticrew M. Developing and evaluating complex interventions: Following considerable development in the field since 2006, MRC and NIHR have jointly commissioned an update of this guidance to be published in 2019. [Internet]. 2006 [cited 2019 Jan 8]. Available from: https://mrc.ukri.org/documents/pdf/complex-interventions-guidance/

32. O'Cathain A, Croot L, Duncan E, Rousseau N, Sworn K, Turner KM, et al. Guidance on how to develop complex interventions to improve health and healthcare. Open Access. 2019;(e029954):9.

33. Farquhar M, Ewing G, Booth S. Using mixed methods to develop and evaluate complex interventions in palliative care research - Morag C Farquhar, Gail Ewing, Sara Booth, 2011. Palliat Med [Internet]. 2011 Aug 1 [cited 2020 Nov 17]; Available from: https://journals.sagepub.com/doi/10.1177/0269216311417919?url_ver=Z39.88-2003&rfr_id=ori%3Arid%3Acrossref.org&rfr_dat=cr_pub++0pubmed

34. Moore GF, Audrey S, Barker M, Bond L, Bonell C, Hardeman W, et al. Process evaluation of complex interventions: Medical Research Council guidance. BMJ [Internet]. 2015 Mar 19 [cited 2020 Nov 5];350. Available from: https://www.bmj.com/content/350/bmj.h1258

35. Thabane L, Ma J, Chu R, Cheng J, Ismaila A, Rios LP, et al. A tutorial on pilot studies: the what, why and how. BMC Med Res Methodol. 2010 Jan 6;10(1):1.

36. Hooper R. Justifying sample size for a feasibility study [Internet]. National Institute for Health Research; 2019 [cited 2020 Nov 5]. Available from: https://www.nihr.ac.uk/documents/nihr-research-for-patient-benefit-rfpb-programme-guidance-on-applying-for-feasibility-studies/20474

37. Lepore SJ, Greenberg MA, Bruno M, Smyth JM. The writing cure: How expressive writing promotes health and emotional well-being. In: The writing cure: How expressive writing promotes health and emotional well-being. Washington, DC, US: American Psychological Association; 2002. p. xii, 313. (Lepore SJ, Smyth JM, editors. The writing cure: How expressive writing promotes health and emotional well-being).

38. Cameron LD, Jago L. Emotion regulation interventions: A common-sense model approach. Br J Health Psychol. 2008;13(2):215–21.

39. Koole SL. The psychology of emotion regulation: An integrative review. Cogn Emot. 2009 Jan 1;23(1):4–41.

40. J Q, M M, Jj G. Positive interventions: An emotion regulation perspective [Internet]. Vol. 141, Psychological bulletin. Psychol Bull; 2015 [cited 2020 Nov 6]. Available from: https://pubmed.ncbi.nlm.nih.gov/25621978/?dopt=Abstract

For peer review only - http://bmjopen.bmj.com/site/about/guidelines.xhtml

41. Nicholls S. Beyond Expressive Writing: Evolving Models of Developmental Creative Writing. J Health Psychol. 2009 Mar;14(2):171–80.

42. Smyth JM, Pennebaker JW. Exploring the boundary conditions of expressive writing: In search of the right recipe. Br J Health Psychol. 2008 Feb;13(1):1–7.

43. Sloan DM, Marx BP. Taking Pen to Hand: Evaluating Theories Underlying the Written Disclosure Paradigm. Clin Psychol Sci Pract. 2004;11(2):121–37.

44. Pennebaker JW. Theories, Therapies, and Taxpayers: On the Complexities of the Expressive Writing Paradigm. Clin Psychol Sci Pract. 2004;11(2):138–42.

45. Hagger M, Luszczynska A. Implementation intention and action planning interventions in health contexts: state of the research and proposals for the way forward. Appl Psychol Health Well-Being. 2014 Mar;6(1):1–47.

46. Harris P, Taylor R, Minor BL, Elliott V, Fernandez M, O'Neal L, et al. The REDCap consortium: Building an international community of software platform partners. J Biomed Inform. 2019 Jul 1;95:103208.

47. Harris P, Taylor R, Thielke R, Payne J, Gonzalez N, Conde J. Research electronic data capture (REDCap) - A metadata-driven methodology and workflow process for providing translational research informatics support. J Biomed Inf. 2009;42(2):377–81.

48. Murtagh FE, Ramsenthaler C, Firth A, Groeneveld EI, Lovell N, Simon ST, et al. A brief, patient- and proxy-reported outcome measure in advanced illness: Validity, reliability and responsiveness of the Integrated Palliative care Outcome Scale (IPOS). Palliat Med. 2019 Sep;33(8):1045–57.

49. Kroenke K, Spitzer RL, Williams JBW, Löwe B. The Patient Health Questionnaire Somatic, Anxiety, and Depressive Symptom Scales: a systematic review. Gen Hosp Psychiatry. 2010 Jul;32(4):345–59.

50. Chilcot J, Rayner L, Lee W, Price A, Goodwin L, Monroe B, et al. The factor structure of the PHQ-9 in palliative care. J Psychosom Res. 2013 Jul 1;75(1):60–4.

51. Kroenke K, Spitzer RL, Williams JBW. The PHQ-9. J Gen Intern Med. 2001 Sep;16(9):606–13.

52. Spitzer RL, Kroenke K, Williams JBW, and the Patient Health Questionnaire Primary Care Study Group. Validation and Utility of a Self-report Version of PRIME-MDThe PHQ Primary Care Study. JAMA. 1999 Nov 10;282(18):1737–44.

53. Spitzer RL, Kroenke K, Williams JBW, Löwe B. A Brief Measure for Assessing Generalized Anxiety Disorder: The GAD-7. Arch Intern Med. 2006 May 22;166(10):1092–7.

54. Snyder E, Cai B, DeMuro C, Morrison MF, Ball W. A New Single-Item Sleep Quality Scale: Results of Psychometric Evaluation in Patients With Chronic Primary Insomnia and Depression. J Clin Sleep Med JCSM Off Publ Am Acad Sleep Med. 2018 Nov 15;14(11):1849–57.

55. Buysse D, Reynolds C, Monk T, Berman S, Kupfer D. The Pittsburgh Sleep Quality Index: a new instrument for psychiatric practice and research [Internet]. Vol. 28, Psychiatry research. Psychiatry Res; 1989 [cited 2020 Nov 9]. Available from: https://pubmed.ncbi.nlm.nih.gov/2748771/

For peer review only - http://bmjopen.bmj.com/site/about/guidelines.xhtml

56. Kupeli N, Schmidt UH, Campbell IC, Chilcot J, Roberts CJ, Troop NA. The impact of an emotionally expressive writing intervention on eating pathology in female students. Health Psychol Behav Med. 2018 Jan 1;6(1):162–79.

57. Sque M, Walker W, Long-Sutehall T. Research with bereaved families: A framework for ethical decision-making. Nurs Ethics. 2014 Dec 1;21(8):946–55.

58. Sekhon M, Cartwright M, Francis JJ. Acceptability of healthcare interventions: an overview of reviews and development of a theoretical framework. BMC Health Serv Res. 2017 Jan 26;17(1):88.

59. Moore G, Audrey S, Barker M, Bonell C, Hardeman W, Moore L, et al. Process evaluation of complex interventions: UK Medical Research Council (MRC) guidance [Internet]. MRC Population Health Science Research Network; [cited 2020 Nov 5]. Available from: https://mrc.ukri.org/documents/pdf/mrc-phsrn-process-evaluation-guidance-final/

60. Nvivo [Internet]. QSR International; Available from: https://www.qsrinternational.com/nvivo-qualitative-data-analysis-software/home

61. IBM SPSS Statistics for Windows. IBM Corp; 2016.

62. Tausczik YR, Pennebaker JW. The Psychological Meaning of Words: LIWC and Computerized Text Analysis Methods. J Lang Soc Psychol. 2010 Mar;29(1):24–54.

63. Roberts K, Dowell A, Nie J-B. Attempting rigour and replicability in thematic analysis of qualitative research data; a case study of codebook development. BMC Med Res Methodol. 2019 Mar 28;19(1):66.

64. Hennink MM, Kaiser BN, Marconi VC. Code Saturation Versus Meaning Saturation: How Many Interviews Are Enough? Qual Health Res. 2017 Mar 1;27(4):591–608.

65. UK Data Service. ReShare: archiving and sharing research data [Internet]. [cited 2020 Nov 11]. Available from: https://reshare.ukdataservice.ac.uk/

For peer review only

For peer review only - http://bmjopen.bmj.com/site/about/guidelines.xhtml

Expression Session topics

- Follow the general instructions in the yellow box below for each Expression Session.
- The topics for each of the three Expression Sessions are described on the next page. Feel free to change the order or repeat a session of your choice if you wish. These topics are just a guide.
- At the beginning of each session, set your timer and try to write in response to the suggested topic for at least 20 minutes. If you become too tired to continue, feel free to pause and pick back up again when you feel able. If at the end of 20 minutes you feel you have more to write, set the timer for another 10 minutes and keep going until you feel ready to stop. We would not recommend you write for longer than 30 minutes in total though.

General Instructions

Keep these in mind throughout each Expression Session

- As you are writing, try to remember and describe the thoughts and emotions you felt during each experience.
- Some of the moments you describe might be painful or difficult to remember. As you describe these experiences, try to write from the perspective of someone who is accepting and understanding of your emotions, and consider how other people may feel the same way when in similar situations. Try to write with kindness and concern for yourself in mind. It might help to think about what you would say to a friend if they were in your shoes, or what a friend might say to you.
- At the end of each session, try to finish by writing a few sentences focussing on expressing warmth and kindness for yourself and your feelings.

Don't worry about spelling, sentence structure, or grammar.

Topic 1: Tell the story of your illness – from when you were diagnosed to now

- What do you think of as the 'key' or 'most important' moments in your illness journey?
- Have there been any particularly difficult moments? How did you feel at the time? How do you feel about that moment now?
- Taking a step back and looking at the bigger picture, try to write a few sentences expressing understanding for the way you feel about those difficult times, and consider how this is something that anyone may feel.

Topic 2: Share your thoughts and feelings about being referred to the hospice

- What does the word *hospice* make you think of? How did you feel when you were first referred? How do you feel now? Have your feelings about receiving care from the hospice changed over time? Are there any good points? Any bad points?
- Taking a step back, can you understand why you may have felt or feel this way about hospice care?
- How do you think your feelings compare to other people who also receive hospice care? What would you say to somebody who is being referred to a hospice?

Topic 3: Tell the story of one or more of your happiest memories

- Think of the most wonderful experiences in your life. What have been some of the happiest or most significant moments for you? This could be a big event, a beautiful scene, falling in love or listening to music. Anything that brought you joy or felt meaningful to you. Describe those moments. How did you feel then? How does it make you feel thinking about them?
- Why do you think these moments feel so significant for you?

Remember to finish each session by writing a few sentences expressing warmth and kindness for yourself and your feelings.

CONSORT 2010 checklist of information to include when reporting a pilot or feasibility trial*

Section/Topic	Item No	Checklist item	Reported on page No
**Title and abstract**			
	1a	Identification as a pilot or feasibility randomised trial in the title	N/A
	1b	Structured summary of pilot trial design, methods, results, and conclusions (for specific guidance see CONSORT abstract extension for pilot trials)	2
**Introduction**			
Background and objectives	2a	Scientific background and explanation of rationale for future definitive trial, and reasons for randomised pilot trial	4-5
	2b	Specific objectives or research questions for pilot trial	5
**Methods**			
Trial design	3a	Description of pilot trial design (such as parallel, factorial) including allocation ratio	5
	3b	Important changes to methods after pilot trial commencement (such as eligibility criteria), with reasons	N/A
Participants	4a	Eligibility criteria for participants	6
	4b	Settings and locations where the data were collected	6, 9-10
	4c	How participants were identified and consented	6-7
Interventions	5	The interventions for each group with sufficient details to allow replication, including how and when they were actually administered	7-9
Outcomes	6a	Completely defined prespecified assessments or measurements to address each pilot trial objective specified in 2b, including how and when they were assessed	9-15
	6b	Any changes to pilot trial assessments or measurements after the pilot trial commenced, with reasons	N/A
	6c	If applicable, prespecified criteria used to judge whether, or how, to proceed with future definitive trial	N/A
Sample size	7a	Rationale for numbers in the pilot trial	6
	7b	When applicable, explanation of any interim analyses and stopping guidelines	N/A
Randomisation:			
Sequence generation	8a	Method used to generate the random allocation sequence	N/A
	8b	Type of randomisation(s); details of any restriction (such as blocking and block size)	N/A
Allocation concealment mechanism	9	Mechanism used to implement the random allocation sequence (such as sequentially numbered containers), describing any steps taken to conceal the sequence until interventions were assigned	N/A

For peer review only

Implementation	10	Who generated the random allocation sequence, who enrolled participants, and who assigned participants to interventions	N/A
Blinding	11a	If done, who was blinded after assignment to interventions (for example, participants, care providers, those assessing outcomes) and how	N/A
	11b	If relevant, description of the similarity of interventions	N/A
Statistical methods	12	Methods used to address each pilot trial objective whether qualitative or quantitative	10-13
**Results**			
Participant flow (a diagram is strongly recommended)	13a	For each group, the numbers of participants who were approached and/or assessed for eligibility, randomly assigned, received intended treatment, and were assessed for each objective	N/A
	13b	For each group, losses and exclusions after randomisation, together with reasons	N/A
Recruitment	14a	Dates defining the periods of recruitment and follow-up	N/A
	14b	Why the pilot trial ended or was stopped	N/A
Baseline data	15	A table showing baseline demographic and clinical characteristics for each group	N/A
Numbers analysed	16	For each objective, number of participants (denominator) included in each analysis. If relevant, these numbers should be by randomised group	N/A
Outcomes and estimation	17	For each objective, results including expressions of uncertainty (such as 95% confidence interval) for any estimates. If relevant, these results should be by randomised group	N/A
Ancillary analyses	18	Results of any other analyses performed that could be used to inform the future definitive trial	N/A
Harms	19	All important harms or unintended effects in each group (for specific guidance see CONSORT for harms)	N/A
	19a	If relevant, other important unintended consequences	N/A
**Discussion**			
Limitations	20	Pilot trial limitations, addressing sources of potential bias and remaining uncertainty about feasibility	N/A
Generalisability	21	Generalisability (applicability) of pilot trial methods and findings to future definitive trial and other studies	N/A
Interpretation	22	Interpretation consistent with pilot trial objectives and findings, balancing potential benefits and harms, and considering other relevant evidence	N/A
	22a	Implications for progression from pilot to future definitive trial, including any proposed amendments	N/A
**Other information**			
Registration	23	Registration number for pilot trial and name of trial registry	N/A
Protocol	24	Where the pilot trial protocol can be accessed, if available	N/A
Funding	25	Sources of funding and other support (such as supply of drugs), role of funders	21
	26	Ethical approval or approval by research review committee, confirmed with reference number	2,6

For peer review only

Citation: Eldridge SM, Chan CL, Campbell MJ, Bond CM, Hopewell S, Thabane L, et al. CONSORT 2010 statement: extension to randomised pilot and feasibility trials. BMJ. 2016;355.

*We strongly recommend reading this statement in conjunction with the CONSORT 2010, extension to randomised pilot and feasibility trials, Explanation and Elaboration for important clarifications on all the items. If relevant, we also recommend reading CONSORT extensions for cluster randomised trials, non-inferiority and equivalence trials, non-pharmacological treatments, herbal interventions, and pragmatic trials. Additional extensions are forthcoming: for those and for up to date references relevant to this checklist, see www.consort-statement.org.

For peer review only

Reporting checklist for qualitative study.

Based on the SRQR guidelines.

Instructions to authors

Complete this checklist by entering the page numbers from your manuscript where readers will find each of the items listed below.

Your article may not currently address all the items on the checklist. Please modify your text to include the missing information. If you are certain that an item does not apply, please write "n/a" and provide a short explanation.

Upload your completed checklist as an extra file when you submit to a journal.

In your methods section, say that you used the SRQRreporting guidelines, and cite them as:

O'Brien BC, Harris IB, Beckman TJ, Reed DA, Cook DA. Standards for reporting qualitative research: a synthesis of recommendations. Acad Med. 2014;89(9):1245-1251.

		Reporting Item	Page Number
**Title**			
	#1	Concise description of the nature and topic of the study identifying the study as qualitative or indicating the approach (e.g. ethnography, grounded theory) or data collection methods (e.g. interview, focus group) is recommended	1
**Abstract**			
	#2	Summary of the key elements of the study using the abstract format of the intended publication; typically includes background, purpose, methods, results and conclusions	2
**Introduction**			
Problem formulation	#3	Description and signifcance of the problem / phenomenon studied: review of relevant theory and empirical work; problem statement	4-5

For peer review only

Purpose or research question	#4	Purpose of the study and specific objectives or questions	5
**Methods**			
Qualitative approach and research paradigm	#5	Qualitative approach (e.g. ethnography, grounded theory, case study, phenomenolgy, narrative research) and guiding theory if appropriate; identifying the research paradigm (e.g. postpositivist, constructivist / interpretivist) is also recommended; rationale. The rationale should briefly discuss the justification for choosing that theory, approach, method or technique rather than other options available; the assumptions and limitations implicit in those choices and how those choices influence study conclusions and transferability. As appropriate the rationale for several items might be discussed together.	5
Researcher characteristics and reflexivity	#6	Researchers' characteristics that may influence the research, including personal attributes, qualifications / experience, relationship with participants, assumptions and / or presuppositions; potential or actual interaction between researchers' characteristics and the research questions, approach, methods, results and / or transferability	15
Context	#7	Setting / site and salient contextual factors; rationale	6
Sampling strategy	#8	How and why research participants, documents, or events were selected; criteria for deciding when no further sampling was necessary (e.g. sampling saturation); rationale	6 - 7
Ethical issues pertaining to human subjects	#9	Documentation of approval by an appropriate ethics review board and participant consent, or explanation for lack thereof; other confidentiality and data security issues	6 - 7, 20
Data collection methods	#10	Types of data collected; details of data collection procedures including (as appropriate) start and stop dates of data collection and analysis, iterative	5, 6, 9-15

For peer review only

		process, triangulation of sources / methods, and modification of procedures in response to evolving study findings; rationale	
Data collection instruments and technologies	#11	Description of instruments (e.g. interview guides, questionnaires) and devices (e.g. audio recorders) used for data collection; if / how the instruments(s) changed over the course of the study	9-15
Units of study	#12	Number and relevant characteristics of participants, documents, or events included in the study; level of participation (could be reported in results)	6
Data processing	#13	Methods for processing data prior to and during analysis, including transcription, data entry, data management and security, verification of data integrity, data coding, and anonymisation / deidentification of excerpts	9-10, 15 - 17, 20
Data analysis	#14	Process by which inferences, themes, etc. were identified and developed, including the researchers involved in data analysis; usually references a specific paradigm or approach; rationale	15-19
Techniques to enhance trustworthiness	#15	Techniques to enhance trustworthiness and credibility of data analysis (e.g. member checking, audit trail, triangulation); rationale	15-17
**Results/findings**			
Syntheses and interpretation	#16	Main findings (e.g. interpretations, inferences, and themes); might include development of a theory or model, or integration with prior research or theory	N/A
Links to empirical data	#17	Evidence (e.g. quotes, field notes, text excerpts, photographs) to substantiate analytic findings	N/A
**Discussion**			
Intergration with prior work, implications, transferability and contribution(s) to the field	#18	Short summary of main findings; explanation of how findings and conclusions connect to, support, elaborate on, or challenge conclusions of earlier scholarship; discussion of scope of application /	N/A

For peer review only

		generalizability; identification of unique contributions(s) to scholarship in a discipline or field	
Limitations	#19	Trustworthiness and limitations of findings	N/A
**Other**			
Conflicts of interest	#20	Potential sources of influence of perceived influence on study conduct and conclusions; how these were managed	21
Funding	#21	Sources of funding and other support; role of funders in data collection, interpretation and reporting	21

None The SRQR checklist is distributed with permission of Wolters Kluwer © 2014 by the Association of American Medical Colleges. This checklist can be completed online using https://www.goodreports.org/, a tool made by the EQUATOR Network in collaboration with Penelope.ai

For peer review only

Good Reporting of A Mixed Methods Study (GRAMMS) checklist[1]

Guideline	Section: page
Describe the justification for using a mixed methods approach to the research question	Methods and analysis (Design): page 5
Describe the design in terms of the purpose, priority and sequence of methods	Methods and analysis (Design): page 5; (Data collection): page 9 - 15
Describe each method in terms of sampling, data collection and analysis	Methods and analysis (Participants and recruitment): page 6 – 7;(Data collection and analysis): page 9 - 19
Describe where integration has occurred, how it has occurred and who has participated in it	Methods and analysis (Data analysis – primary objective): page 15, Table 3)
Describe any limitation of one method associated with the present of the other method	Methods and analysis (Design): page 5
Describe any insights gained from mixing or integrating methods	Tbc – protocol therefore results not reported (see Methods [design section] for justification / aims)

1. O'Cathain A, Murphy E, Nicholl J. The quality of mixed methods studies in health services research. *J Health Serv Res Policy.* 2008;13: 92-98.

For peer review only
